# Screening putative polyester polyurethane degrading enzymes with semi-automated cell-free expression and nitrophenyl probes

**DOI:** 10.1093/synbio/ysae005

**Published:** 2024-02-13

**Authors:** Afrin Ahsan, Dominique Wagner, Vanessa A Varaljay, Victor Roman, Nancy Kelley-Loughnane, Nigel F Reuel

**Affiliations:** Department of Chemical and Biological Engineering, Iowa State University, Ames, IA, USA; Materials and Manufacturing Directorate, Air Force Research Laboratory, Wright-Patterson AFB, OH, USA; UES Inc., Dayton, OH, USA; Materials and Manufacturing Directorate, Air Force Research Laboratory, Wright-Patterson AFB, OH, USA; Materials and Manufacturing Directorate, Air Force Research Laboratory, Wright-Patterson AFB, OH, USA; Materials and Manufacturing Directorate, Air Force Research Laboratory, Wright-Patterson AFB, OH, USA; Department of Chemical and Biological Engineering, Iowa State University, Ames, IA, USA

**Keywords:** plastic degradation, enzymes, cell free, high throughput screening

## Abstract

Cell-free expression (CFE) has shown recent utility in prototyping enzymes for discovery efforts. In this work, CFE is demonstrated as an effective tool to screen putative polyester polyurethane degrading enzyme sequences sourced from metagenomic analysis of biofilms prospected on aircraft and vehicles. An automated fluid handler with a controlled temperature block is used to assemble the numerous 30 µL CFE reactions to provide more consistent results over human assembly. In sum, 13 putative hydrolase enzymes from the biofilm organisms as well as a previously verified, polyester-degrading cutinase were expressed using in-house *E. coli* extract and minimal linear templates. The enzymes were then tested for esterase activity directly in extract using nitrophenyl conjugated substrates, showing highest sensitivity to shorter substrates (4-nitrophenyl hexanoate and 4-nNitrophenyl valerate). This screen identified 10 enzymes with statistically significant activities against these substrates; however, all were lower in measured relative activity, on a CFE volume basis, to the established cutinase control. This approach portends the use of CFE and reporter probes to rapidly prototype, screen and design for synthetic polymer degrading enzymes from environmental consortia.

Graphical Abstract

**Figure:**
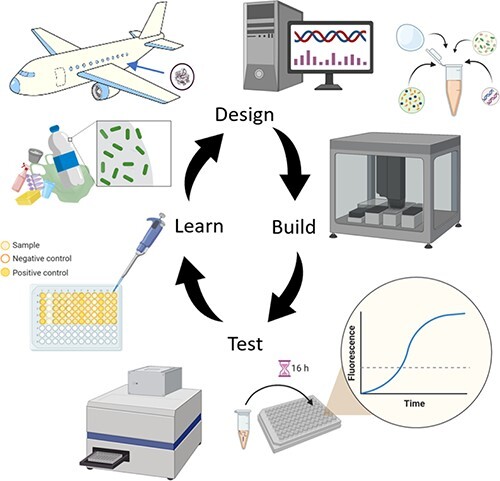

## 1. Introduction

Bio-corrosion is a costly threat to modern-day infrastructure and transportation networks but may also, simultaneously, hold key information to sustainable disposal of recalcitrant, synthetic materials such as plastics. Bio-corrosion refers to the deterioration of metal or nonmetal materials caused by the influence of microorganisms typically growing as a biofilm (1). It is observed in many environments and media including seawater, freshwater, soils, foodstuffs, demineralized water, sewage, aircraft petrol, human plasma and process chemicals (2). These microbes can be studied to determine how to best protect against these destructive effects and can also provide a rich source of prospected enzymes for the degradation of materials such as plastics.

Plastics are a staple of modern life. Starting with natural polymers, horn, waxes, rubbers and resins (3), progressing to the discovery of vulcanized rubber and polystyrene (PS) in 1839 (4), they have progressed to be their own large, synthetic material class. As plastics are lightweight and more resistant to chemical, physical and biological degradation, they have many functional usages (5). However, this very resistance (6) has led to massive accumulation of plastics. Micro- and nano-sized plastic particles have spread in terrestrial and aquatic ecosystems causing widespread health concerns (7).

Plastic waste is now a global concern, and much research is being done on potential routes of remediation. Using microbial or enzymatic degradation of plastics is a promising method to depolymerize waste plastics into virgin monomers to recycle or upconvert them (8). The degradation occurs by enzymes present in or secreted by microbial cells. Low crystalline and some low-density polymers of polyethylene terephthalate, (PET), ester-based PUR and oligomers of PA are biodegradable by hydrolases (9). The enzymatic degradation occurs in two stages: adsorption of enzymes on the polymer surface, followed by hydro-peroxidation/hydrolysis of the bonds (10). This is typically done extracellularly. They facilitate the addition of a water molecule to the polymer chain, resulting in the cleavage of the carbon bond and then formation of smaller fragments (11). Then, smaller subunits can be assimilated into the microbial cell for further enzymatic degradation and then release metabolic products like CO_2_, H_2_O, CH_4_ and N_2_ (12). More than 90 microorganisms like bacteria and fungi can degrade petroleum-based plastics (13); these have been isolated from soil, sea water, activated sludge, etc. and hydrolyze different polymers (14). Metagenomic DNA from biocorrosion films can be sequenced to provide a large database of putative enzymes. However, one limitation to the field is rapidly prototyping these putative enzymes and obtaining experimental sequence to function data.

Cell-free expression (CFE) is an established tool for rapid protein prototyping, with applications in metabolic engineering, therapeutic development and sensor design (15, 16). Briefly, CFE uses extracted biomolecular machinery to produce proteins without using living cells. Cell extracts are used that contain essential transcription–translation machinery; the extract is also supplemented to further extend reaction time. DNA templates are added to the extract and the expression proceeds *in vitro* (17). One limitation of CFE is the inherent variability in experimental replicates (18), which can be partially mitigated through improved extract and supplement preparation and improved manual handling of small volumes (19).

Automation of CFE can further improve consistency. As examples, a BRAVO liquid-handling robot (Agilent Technologies) was used to map physiochemical landscapes to maximize biosynthesis yields (20). The Echo 525 Acoustic Liquid Handler (Beckman Coulter Life Sciences, San Jose, CA) has been used to further reduce sample volume and increase throughput (21) and to explore regulatory mechanisms for T7 RNAP-driven expression; a rapid and cost-effective method was developed to characterize engineered T7-based transcription factors using cell-free protein synthesis and the liquid handler (22). Although small-volume, acoustic-based liquid handlers (e.g. Echo) are becoming more prevalent in the field, they are not well-equipped to produce 10–100 µL reactions that are necessary, in some cases, to produce enough protein for functional analysis. Moreover, the cost point of these liquid handlers is prohibitive to many users. Here, we demonstrate the use of a low-cost, automated pipettor (Opentrons, OT-2) to overcome these limitations and screen plastic degrading enzymes.

**Figure 1.**
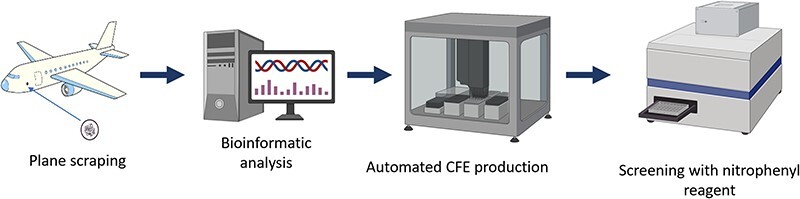
Overview of workflow (left to right): aircraft and truck biofilms are collected and sequenced, bioinformatic analysis is performed to down select to panel of enzymes to test from the genomic database, these are produced as proteins using automated CFE and finally degradation extent is screened using a nitrophenyl reagents directly in the completed CFE reaction.

In this work, putative hydrolases were mined from genomic data of microbes found to grow on aircraft and trucks (23–25) (Figure 1). The target substrate in this case is polyester polyurethane which is used as a topcoat finish for interior use on high performance aircraft. A panel of 13 unique enzymes was identified for testing on polyester degradation activity using current bioinformatic workflows. These enzymes were expressed using semi-automated CFE and tested with chromogenic probes (nitrophenyl substrates) to rank order the polyester degrading ability of each sequence, per reaction volume. We then discuss limitations of this approach and advances necessary to make it a more relevant screening method.

## 2. Materials and methods

### 2.1 Genomic assessment

**Figure 2.**
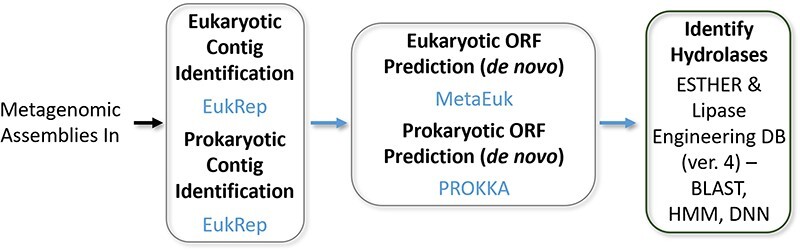
Identification of putative hydrolases from prospected organism metagenomic data.

As described in more detail (24, 25), microbial communities residing on polymer-coated interior surfaces of military aircraft and trucks were sourced and sequenced. These communities consisted of fungi and bacteria and were collected to characterize microbial degradation of polymers. Metagenomic assemblies were classified into eukaryotic and prokaryotic contigs using EukRep (26), and then eukaryotic open reading frames (ORFs) were predicted with MetaEuk (27) and prokaryotic ORFs were predicted using Prokka (28). Using a combination of sequence identification tools (BLAST (29), HMM (30) and DeepGOPlus (31)) and alpha/beta fold hydrolase-specific reference databases (ESTER (32) and Lipase Engineering Database (33)), we identified putative hydrolases (Figure 2). A subset of 13 hydrolases that included several cutinases (known to degrade polyesters (34)) and other polymer substrates, as well as representatives from both fungal and bacterial species, were selected for testing on the automated CFE platform (see Supplement 1 for sequences).

### 2.2 Opentrons accuracy and precision measurements

Fluid handling accuracy experiments were conducted by adding 1 µL water (at room temperature) and assessing level of replicate variance through mass measurement. The mass of an empty PCR tube was measured, 1 μL water was added with Opentrons, and the final mass was measured. The same operations were performed nine independent times using the same tip as well as with changing the tip at each step. To measure precision of automated CFE reaction preparation from same stock tubes of reagents, 1 μL of dye (food coloring) was added to 14 μL of water at room temperature. In total, 48 readings were taken using the same tip and changed tip at each step and they were done both manually and with the OT-2. The absorbance of each well was then measured using Synergy Neo2 HTS Multi-Mode Microplate Reader (BioTek) at 420 nm. Statistical assessment of variance was done with a two-sample F test. An F test can determine if the variance of one population is significantly different from variance of another and determine the associated *P*-value (35).

### 2.3 CFE reactions

**Figure 3.**
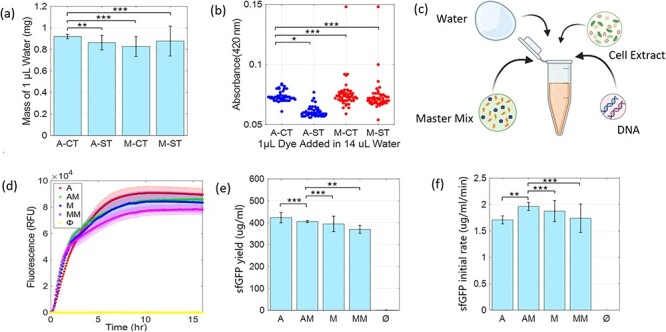
Benchmarking automated assembly. (a) Accuracy check using mass of 1 μL water that was added manually and with OT-2 using same versus change tip protocols. In the figure, A and M are automated and manual process, respectively; CT and ST are changed tip and same tip, respectively (*n* = 9 samples). (b) Precision measured by measuring absorbance when 1 μL dye was added to 14 μL water (same conditions as (a), *n* = 48)). (c) Four components added in a CFE reaction. (d) sfGFP reporter protein dynamic fluorescence response in CFE reactions done by automated and manual methods; A, AM, M and MM stand for automated transfer, automated transfer and mix, manual transfer and manual transfer and mix, respectively. (e) End point yield of sfGFP for the CFE reactions with standard deviation (*n* = 5). (f) sfGFP production rate at first 2.5 h of the CFE reactions (*n* = 5). Error bars for all plots show one standard deviation. The star marks show *P*-values from testing significance of variance difference between methods through two sample F-test, indicating *P*-value with stars (*P* < 0.05, (*), *P* < 0.01, (**), *P* < 0.001, (***)).

In the CFE reactions, four components are added: cell extract, supplement mix, DNA template and water (Figure 3c). These components were added to the reaction well plate manually or with an automatic fluid handler (OT-2). For our experiment, we used *E. coli* (BL21 DE3 star) cell extract, PANOx-SP system as supplement (with 57 mM HEPES buffer of pH 7, 1.2 mM ATP, 0.85 mM GMP, 0.85 mM CMP, 0.85 mM UMP, 33 mM PEP, 34 μg/ml folinic acid, 171 μg/ml *E. coli* tRNA, 2 mM 20 amino acids, 0.33 mM NAD, 0.26 mM CoA, 175 mM potassium glutamate, 10 mM ammonium glutamate, 16 mM magnesium glutamate, 2.7 mM potassium oxalate, 1 mM putrescine, 1.5 mM spermidine) and a linear DNA template at 5 nM (36). The cell extract was produced following the methodology outlined in our lab’s prior publication on scalable extract (19). A 96 clear well plate (Corning® 96 Well White PS microplate) was used to contain the reactions. Each well contained 30 μL of reaction solution composed of 10 μL of supplement mix, 7.2 μL of cell extract (Bl21 DE3 star), 5 nM of linear template and balance of volume as water. In the control experiments, the components were 10 μL of supplement mix, 7.2 μL of cell extract (Bl21 DE3 star) and 12.8 μL of water. The DNA sequences of the enzymes were obtained using the IDT codon optimization tool for *E. coli*. Integrated DNA Technologies (IDT) provided the software IDT Codon Optimization Tool to facilitate the optimization of DNA sequences for enhanced protein expression in various host organisms. Using the tool, the researchers can design synthetic DNA sequences that are adapted to the preferred codon usage of the target organism, alleviating issues related to codon bias, tRNA availability, mRNA stability, which can substantially increase protein production levels (37, 38). The enzyme sequence was inserted into the protein sequence location of our minimal expression template with primer binding sites, restriction enzyme sites, a T7 promoter, a ribosome bindingsite, a start codon, T7 terminator and was ordered from IDT as a complete gene block. After receiving the gene fragment, it was suspended and went through traditional linear amplification using OneTaq kit. Protocols supplied by OneTaq were followed for the amplification. The resulting linear template (LET) was then purified using Zymo purification kit (D4004) and eluted in 50 μl of water. The concentration was determined via 260/280 nm absorbance measurements with a Synergy NEO2 multimode reader (BioTek, Winooski, VT) (39, 40) (all sequences used in this study are available in Supplement 1). In the automated experiments, master vials of extract, supplement mix and DNA were used to construct five independent replicates. Here, the reagents were transferred and mixed by pipette of OT-2. The reagents were held at 4°C to reduce the deterioration of quality of the reagents during setup using the OT-2 temperature module. In manual experiments, five replicates were also prepared for each sample to account for the biological variability encountered in CFE experiments. After mixing the components, the wells were surrounded by water and the plate was covered to avoid drying (40). The plate was monitored in a plate reader for 16 h at 30°C with orbital shaking to measure dynamic expression of the protein. This experiment was done to measure variance in production of a model protein, superfolder green fluorescent protein (sfGFP) by automated versus manual reaction preparation. For the nitrophenyl testing with 13 putative enzyme experiments, the CFE reaction was run for 5 h at 30°C on a thermomixer block. After the CFE reaction was complete, the samples were tested with nitrophenyl probes as described next.

### 2.4 Nitrophenyl probe experiments

The completed CFE reactions containing enzyme were initially tested with 4-nitrophenyl hexanoate (4-NPH), a colorigenic esterase probe demonstrated in literature (41). The cleavage of the p-nitrophenyl ester (42) releases the product, 4-NP (4-nitrophenol) and imparts a bright yellow color to the solution, which can be measured at 405 nm (42, 43). To prepare the colorogenic substrates, a stock solution of 2.5 mM 4-nitrophenyl hexanoate (4NPH) was made in dimethylformamide (DMF) prior to use (light sensitive reagent) (41). After the CFE reaction was complete and maintained at 25°C, 70 μL of phosphate buffered saline (1× PBS; pH 8.0) was added to the reaction well to maintain constant pH during the assay, which is critical for enzyme activity measurements (44); this also diluted the sample and reduced background effect of CFE reagents interacting with the nitrophenyl probe. Then, 10 μL of 2.5 mM of 4NPH was added to the wells of expressed enzyme (final probe concentration = 0.227 mM). The substrate was added using an automated dispenser included in the plate reader. The change in absorbance was measured after 1 min of adding the probe as the cleaving activity of the ester was observed to happen quickly and this reduced background effect of CFE components interacting with the probe, which was observed in longer incubation times (15 and 30 min) with sfGFP control reactions (Supplement 7).

The change in absorbance at 405 nm was observed with the spectral scan option in the Synergy Neo2 HTS Multi-Mode Microplate Reader (BioTek). This procedure was conducted with the full panel of putative enzymes against four nitrophenyl substrates with varying carbon chain lengths: 4-nitrophenyl hexanoate (4NPH), 4-nitrophenyl palmitate (4NPP), 4-nitrophenyl valerate (4NPV) and 4-nitrophenyl dodecanoate (4NPD). As a control, wells expressing sfGFP in CFE were tested with the nitrophenyl substrates to check for any nonselective activity from the CFE components (and to cross validate that the expression conditions were good by measuring sfGFP fluorescence).

## 3. Results and discussions

Prior to using CFE for screening a panel of enzymes, we evaluated the accuracy, precision and throughput advantages of a low-cost, automated fluid handler (OT-2), relative to manual reaction assembly, especially with regard to low volume handling. The typical lower volume limit manual addition in a CFE screening experiment is 1 μL, and thus performance is assessed at that level. First, accuracy and level of variance were compared between manual and automated dispensing of 1 μL water and comparing to expected mass (Figure 3a). This experiment showed that automated addition with changed tip between each deposit had closest to expected with average of 0.92 mg (as measured on our balance) and tightest variance with standard deviation of ± 0.021 mg and root mean squared error of 0.085 mg; this is better than all other tested protocols including automated pipetting with the same tip. Two sample F tests between the automated, change-tip method and other methods show a statistically significant decrease in variance. The inaccuracy and variability of the methods that keep the same tip on the OT-2 machine is likely due to variable effects of loading a wetted surface area on repeated use runs. Manual addition has bigger variability likely due to more difficult pipetting technique at such low volumes. When the same tip is used between runs, there is always a certain amount of remaining water inside the tip that causes a difference in pulled liquid volume in the next run because of the change in surface tension. Also, manual pipetting occurs at variable angles and insertion depths causing more variation than automated methods which do not have this limitation.

Next, precision of the automated versus manual additions was compared using 1 μL dye additions to different wells with and without changing the tip, measuring the absorbance caused by the dye, and then assessing variance (Figure 3b). It was observed that the automated dye addition using the changed tip is more precise than the other readings (lowest standard deviation) and variance is significantly lower than the other manual methods (based on F-test, see Figure 3b, also Supplement 5). Based on results of the accuracy and precision tests, we established an OT-2 protocol using Opentrons Protocol Designer BETA software for assembling a CFE reaction (45).

Next, we evaluated reproducibility of CFE reactions with automation provided by the OT-2. The same source of reagents was used for both manual and automated CFE reactions, and expression progress was tracked using fluorescence of sfGFP (Figure 3d, *n* = 5). Reactions were also tested as ‘only transferred’ (no pipette mixing to reduce tip consumption) as well as ‘transferred and mixed’ via pipetting. The end point yield (Figure 3e) and initial production rate at first 2.5 h of the reaction (Figure 3f) were measured. Variation in response is reduced with mixing for both automated and manual additions. Automated addition with mixing has higher GFP production rate (1.96 µg/ml/min) with less deviation (±0.08 µg/ml/min). Moreover, the automated additions showed higher protein yield than manual additions (per end point fluorescence, Supplement 2). An F-test on the results also shows that the automated addition with mixing variance is significantly lower than the manual operations. To quantify the amount of sfGFP that was produced, an sfGFP RFU to mass concentration calibration curve was used (Supplement 2) (19).

These data show that the low-cost, automated fluid handler has less variability than manual operation. It is noted that this is not an exhaustive study and that the manual operation could be improved by screening other users or pipettes; however, screening campaigns would be limited by user to user variability. These data also show that CFE variability is reduced when reagents are mixed by the pipette after transferring to the well plate. Automation also has clear advantages in larger, full well plate campaigns where automation can improve errors caused by pipetting fatigue and user error; tracking specific volumes of reagents to particular wells can be difficult across a full plate with a high chance of adding no reagent or adding more than once to a well. Thus, for the putative hydrolase enzyme testing that follows, the automated method with mixing was used for all experiments.

### 3.1 Prototyping hydrolase enzymes in cell-free reactions

**Figure 4.**
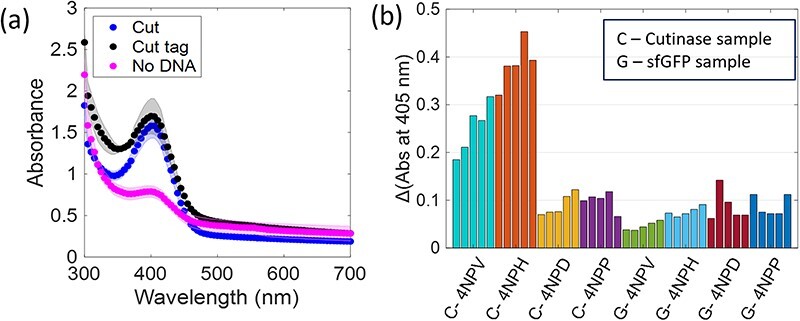
(a) Spectral scan of dilute lysate after incubating with 4-NPH probe, showing peak of cleaved 4-NPH probe at 405 nm after 1 min incubation. (b) Response of cutinase (C) on four different nitrophenyl ester substrates (4NPV, 4NPH, 4NPD and 4NPP) along with sf-GFP (G) control. Each bar shows the increase in absorbance at 405 nm after 1 min incubation. Data are shown for each technical replicate (n = 5).

To first demonstrate that CFE can produce hydrolases that have measurable esterase activity, we used CFE to produce a cutinase (*Papiliotrema laurentii*) with known esterase activity (46) (see Supplement 1 for sequence). The initial experiments revealed much background activity of the cell lysate on the nitrophenyl probe, a common problem with absorbance-based assays. Thus, to limit the effect of background activity, we diluted the completed extract with phosphate-buffered saline (PBS) up to 100 μL, added 10 μL 4-NPH at concentration of 2.5 mM (final concentration = 0.227 mM) using an automated dispenser attached with the plate reader, and then monitored change in absorbance. By screening the plate after adding the probe (incubation of 1 min), we found significant cleavage from the CFE reactions that produced this cutinase, from templates with and without a secretion tag (at 405 nm peak, Figure 4a) as compared to lysate that had no template (negative control).

Next, a panel of four nitrophenyl ester substrates (Table 1) was tested against this cutinase and benchmarked against a CFE control reaction that produced sfGFP to test reproducibility and selectivity. The change in absorbance was measured at 405 nm after 1 min incubation with the probe (ΔAbs_405nm_, Figure 4b). Some background activity from the sfGFP extract is observed, but the change in absorbance caused by the enzyme containing samples is higher than sfGFP. It is also observed that the shorter substrates (4NPV and 4NPH) are more readily cleaved by cutinase than the longer 4NPD and 4NPP. This likely has to do with the accessibility of the enzyme active site to these substrate proxies; if the substrate side chain is too large, it will not fit the catalytic site (47, 48).

**Table 1.** Four nitrophenyl ester substrates with varying carbon chain length used for hydrolase testing

Name	Molecular formula and weight	Chemical structure
4-nitrophenyl valerate (4NPV)	C_11_H_13_NO_4_ 223.23	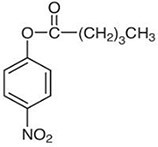
4-nitrophenyl hexanoate (4NPH)	C_12_H_15_NO_4_ 237.25	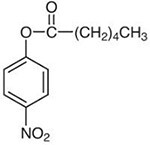
4-nitrophenyl dodecanoate (4NPD)	C_18_H_27_NO_4_ 321.4	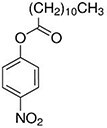
4-nitrophenyl palmitate (4NPP)	*C_22_H_35_NO_4_* 377.5	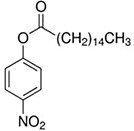

**Figure 5.**
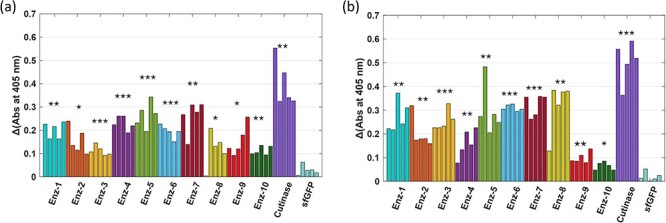
Testing putative enzyme panel against 4-nitrophenyl valerate (a) and 4-nitrophenyl hexanoate (b) substrates. Change in absorbance at 405 nm measured after adding the probes to enzyme or control (cutinase as positive and sfGFP as negative). The statistical significance between sfGFP control and each sample mean is computed with two sample T-test, indicating P-value with stars (P < 0.05, (*), P < 0.01, (**), P < 0.001, (***)).

The 4NPV and 4NPH probes were then used to test the full panel of 13 enzymes identified by metagenome analysis (Supplement 3). Enzymes 1–10 showed significant cleavage (at 405 nm peak) compared to sfGFP control for both substrates (*P*-values tabulated in Supplement 6 and indicated in Figure 5). The positive control cutinase showed maximum signal change for both substrates. The enzymes can show different results at Nitrophenyl test based on substrate; as example, enzyme 8 (Putative Coesterase—*Hortaea werneckii* (Fungi)) is one of the top performers with 4NPH but is ranked eighth with 4NPV. This demonstrates the impact proxy substrates may have in screening efforts, leading to promotion of sequences that might show activity with a given proxy and not to the actual, target substrate. To improve quality of screening results, probes that can operate in small-volume, turbid cell-free reactions with the actual target substrate should be identified, such as recently developed polymer and fluorescent nanoparticle conjugates (49).

Another apparent limitation of this direct screening approach is the inherent biological variability present in CFE reactions, observed in the varied response levels for a given enzyme type (Figure 5). Even with automated CFE assembly, the amount of enzyme expressed can vary and there is inherent variability in the nitrophenyl assays (42, 43) which are very sensitive to dispensed volume and timing. This emphasizes the importance of running sufficient replicates with each test. One method to overcome variability is to express sufficient target enzyme with purification tag and run parallelized separation and purification; however, this comes at cost and increased time.

The approach of CFE-based screening is fast relative to traditional methods of heterologous expression, purification and testing with nitrophenyl substrates or other activity assays. CFE prototyping is a useful tool to identify which sequences from a library exhibit target activity; however, the relative ranking should not be assumed as absolute truth. Follow-on, kinetic studies of top candidates should still be performed. Not only is relative ranking affected by proxy substrate size, as noted above, but there are also differences in template expression. Even though the same amount of DNA template is added to each reaction (5 nM), this does not mean that the same number of active enzymes are produced. This is highly dependent on length and composition of sequence leading to different translational efficiencies (50–52). Uniform expression across a pool of genetic templates has yet to be demonstrated with CFE and is one limitation for rapid prototyping and screening by CFE. Convenient methods to directly quantify the amount of protein being functionally assessed should likewise be developed. Other limitations of this approach include the low level of enzyme production that precludes testing on larger polymer films.

## 4. Conclusion

Herein, we have validated that CFE coupled with an effective reporter probe can be used to provide relative functional information for a panel of bio-prospected, putative enzymes. This is useful for investigators trying to isolate top candidates for deeper study from a large genomic library. In this case, small molecular weight nitrophenyl probes, 4-nitrophenyl valerate and 4-nitrophenyl hexanoate, were found to work directly in diluted CFE reactions in detecting esterase activity. Although these are convenient substrate proxies to polyurethane polyesters, they do not capture the complexity of the actual polyurethane substrate and efforts should continue to develop convenient solution phase, optical reporting probes that transduce actual polymer degradation. Such a probe could be developed from suspending optical nanoparticles in polymer substrate (53) and testing as a colloidal solution or on a porous solid surface. This approach could also solve the inherent limitation of low enzyme yield from small CFE reactions that precludes testing on larger polymer fragments and films. Additionally, continued progress should be made on standardizing CFE reagents and template design such that there are similar translational efficiencies across all expressed products and less variation between replicates. Methods to rapidly measure titer of correctly folded enzymes in the CFE reaction will also help normalize enzyme activity measurements. Notwithstanding these current limitations, automated CFE is a versatile tool to help provide much needed sequence to function data for enzymes. Such standardized data sets can then be used to train machine learning based tools for *de novo* enzyme design.

## Supplementary Material

ysae005_Supp

## Data Availability

The raw data underlying this article will be shared on reasonable request to the corresponding author.
