# Supplementary material for: Screening putative polyester polyurethane degrading enzymes with semi-automated cell-free expression and nitrophenyl probes
Source: Synth Biol (Oxf). 2024 Feb 13;9(1):ysae005. doi: 10.1093/synbio/ysae005 (PMC10898825; doi:10.1093/synbio/ysae005)
Supplement: ysae005_Supp [file ysae005_supp.zip › suppl_data/AutoCFE_PURase_Supplement_V12.pdf]

# Screening Putative Polyester Polyurethane Degrading Enzymes with Semi-Automated Cell Free Expression and Nitrophenyl Probes

Afrin Ahsan[1], Dominique Wagner[2,3], Vanessa A. Varaljay[2], Victor Roman[2], Nancy Kelley-Loughnane[2], Nigel F. Reuel[1]*

1. Department of Chemical and Biological Engineering, Iowa State University, Ames, Iowa
2. Materials and Manufacturing Directorate, Air Force Research Laboratory, Wright-Patterson AFB, Ohio
3. UES Inc., Dayton, Ohio

*Corresponding Author – reuel@iastate.edu

## Table of Contents

Supplement 1. DNA sequences of the used enzymes ........ 1

Supplement 2. Calibration Curve for sfGFP ........ 16

Supplement 3. Test of 13 enzymes with 4-Nitrophenyl Hexanoate substrate ........ 16

Supplement 4. Test of selected 10 enzymes with 4-Nitrophenyl Valerate and 4-Nitrophenyl Hexanoate substrate. ........ 17

Supplement 5. F-test results of automated vs manual experiments ........ 17

Supplement 6. T-test on 4-Nitrophenyl Valerate and 4-Nitrophenyl Hexanoate substrate........ 18

Supplement 7. Observing effect of time with 4-Nitrophenyl Hexanoate probe........ 20

## Supplement 1. DNA sequences of the used enzymes

NOTE: all DNA sequences are submitted as annotated Genbank files as well.

**Table 1:** List of Enzymes used in the experiments

| Enzyme no. | Type | Scientific Name | Group | Source |
|---|---|---|---|---|
| Base Enzyme | Cutinase | *Papiliotrema laurentii* | Fungi | |
| Enzyme 1 | Lipase_3 | *Zymoseptoria brevis* | Fungi | Aircraft |
| Enzyme 2 | Cellulase | *Rachicladosporium antarcticum* | Fungi | Aircraft |
| Enzyme 3 | Cutinase | *Rachicladosporium antarcticum* | Fungi | Aircraft |
| Enzyme 4 | Cutinase | *Hortaea werneckii* | Fungi | Aircraft |
| Enzyme 5 | Cutinase | *Hortaea werneckii* | Fungi | Aircraft |
| Enzyme 6 | Cutinase | *Rachicladosporium antarcticum* | Fungi | Aircraft |
| Enzyme 7 | COesterase | - *Peltaster fructicola* | Fungi | Aircraft |
| Enzyme 8 | COesterase | *Hortaea werneckii* | Fungi | Aircraft |

| | | | | |
|---|---|---|---|---|
| Enzyme 9 | Peptidase_S28 | *Friedmanniomyces simplex* | Fungi | Aircraft |
| Enzyme 10 | Lipase | *Coccomyxa subellipsoidea* | C-169 Fungi | Aircraft |
| Enzyme 11 | Dienelactone hydrolase | *Coleofasciculus sp.* | Bacteria | Truck |
| Enzyme 12 | Alpha/beta hydrolase (unknown substrate) | *Roseomonas sp.* | KE2513-Bacteria | Truck |
| Enzyme 13 | Cutinase | *Talaromyces islandicus* | Fungi | Aircraft |

**Cutinase-** *Papiliotrema laurentii* **(Fungi)**

**Cutinase**:>5307AH_cutinase

# Amino Acids

MKLLSLALTLAALFTSSSTAAPVDIDAVDHLLARAVSPTCSQYTIINTRGTGELQGESSGF
RTMNSRVRAALSGGTIYNTVYAADFSQISTAATQDIIRQVNAGVASDPKRCFILEGYSQG
AAATTNALGQLTGAAFDAVKGVFLIGNPLHQPGLACNVDQNGGTTTRNARGISASFLPG
IPSNWVSKTLDVCIRGDGVCDVAFGVGITAQHLLYPLDSATQSLGTSFITKQLGGSA

**Cutinasew/SecretionTag**:>5307AH_cutinase_amino_acid_secretion_signal(^)

MKLLSLALTLAALFTSSSTA^APVDIDAVDHLLARAVSPTCSQYTIINTRGTGELQGESSG
FRTMNSRVRAALSGGTIYNTVYAADFSQISTAATQDIIRQVNAGVASDPKRCFILEGYSQ
GAAATTNALGQLTGAAFDAVKGVFLIGNPLHQPGLACNVDQNGGTTTRNARGISASFLP
GIPSNWVSKTLDVCIRGDGVCDVAFGVGITAQHLLYPLDSATQSLGTSFITKQLGGSA

# DNA

GTAAAACGACGGCCAGTAGCGCTATTAAAGCTTCGAAATTAATACGACTCACTATA
GGGAGACCACAACGGTTTCCCTCTAGAAATAATTTTGTTTAACTTTAAGAAGGAGAT
ATACATATGATGAAATTACTTAGTCTTGCTCTTACTCTGGCTGCATTGTTCACTAGCA
GCTCGACAGCGGCGCCTGTTGATATCGACGCTGTAGACCACCTGCTTGCTCGCGCTG
TCAGTCCAACCTGTTCTCAATACACTATTATCAACACACGCGGGACAGGTGAACTGC
AGGGGGAGAGCAGCGGCTTTCGTACCATGAACTCCCGTGTTCGTGCGGCCTTAAGCG
GCGGTACTATCTACAACACGGTCTATGCTGCTGATTTCTCACAAATCTCCACTGCAG
CAACCCAAGACATTATCCGTCAGGTCAATGCTGGGGTTGCCTCAGATCCAAAGCGCT
GCTTCATCTTAGAGGGGTATAGTCAGGGTGCTGCAGCGACGACAAATGCTTTAGGTC
AGCTTACCGGGGCAGCGTTTGACGCAGTCAAAGGCGTGTTTTTGATTGGAAATCCCC
TTCACCAACCTGGTCTTGCTTGCAATGTCGATCAAAATGGCGGTACTACGACCCGCA
ACGCCCGTGGCATCTCTGCTTCATTCTTACCTGGGATTCCGTCCAACTGGGTTTCAAA
GACATTGGACGTCTGCATTCGCGGGGATGGGGTTTGCGATGTCGCCTTCGGCGTAGG
TATCACTGCACAGCACTTATTGTATCCTTTGGACTCCGCCACACAATCGTTGGGTACC
TCTTTCATCACAAAACAACTGGGGGGAAGTGCTTAATAAGTCGACCGGCTGCTAACA
AAGCCCGAAAGGAAGCTGAGTTGGCTGCTGCCACCGCTGAGCAATAACTAGCATAA
CCCCTTGGGGCCTCTAAACGGGTCTTGAGGGGTTTTTTGCTGAAAGCGAGACTAAGC
TTTAAACTTCGGGTCATAGCTGTTTCCTG

Needed Genetic Elements by color code:

T7 Promoter

RBS

Start

Protein Sequence –

Stop

T7 Terminator

Circularization site – HindIII Digest

Primer Sequences

**Enzyme 1**

**Lipase_3 (#1) -** *Zymoseptoria brevis* **(Fungi) -** Aircraft

# Amino Acids

>TRINITY_DN21839_c0_g1_i2.p1 type:complete len:623 gc:universal
TRINITY_DN21839_c0_g1_i2:1999-131(-)

MRCLIPSPPPRATASPTAIMMHGSLLLSCLLSITASADAARSPRQRAEQRNPYVILPPGLDNDIGEA
KPPIALEEKEFTLRHIYHHGSHDYPDLHRYMDIPEDANLKVTSDFGATYEPAPRVLRARAASTSM
QRLADRRKSRIDGLLEHAEVHGEAATLPSSAWTVDEVAGPNVTDKETVLSFARMANNAYTHEH
GTGEWQEVGGGFNYTEDFGWEQDGLRGHIFADKTNGTIVIGLKGTSMAIFDGAETTGNDKLND
NLFGSCCCGQGGPYGWKQVCDCMTNTYTCNSTCLVKSLRQKSHYYFAARDLYHNVTERYPNS
DVWMSGHSLGGVVGSLLGLTYGLPVMTFEAYPDAMAASRLGLPTPPGYRIGAHQSRPCTGIHH
YGHTADPIFMGTCNAATSLCTIAGYALQSQCHTGRTCIYDTVGDLGWRTGVGTHRIVNVIKDVIE
KYDTVPSCEEDFECQDCFNWKFFESNSSETTTSSSKPSSTTSQTRTETCKTPGWWGCLDETTTPPT
PTTTMTSTLTTSSCKTPGWFGCKDPTTMTLTTTITSPTAAPAPIVTTTSSTPVTTTSTSLASPSSSVA
SSMSTCKTPGWFGCKDPTSTVSSNTGSSTAAAATYKEGLR*

# DNA

GTAAAACGACGGCCAGTAGCGCTATTAAAGCTTCGAAATTAATACGACTCACTATAGGGAG
ACCACAACGGTTTCCCTCTAGAAATAATTTTGTTTAACTTTAAGAAGGAGATATACATATGA
TGCGTTGTTTAATCCCATCTCCGCCGCCTCGCGCCACTGCCTCCCCGACGGCAATCATGATGC
ACGGTTCCCTTCTTTTAAGTTGTCTTCTCTCTATTACGGCCTCTGCCGACGCCGCTCGTTCTCC
GCGGCAACGGGCCGAGCAGCGCAACCCATATGTTATCCTCCCGCCTGGTTTAGACAATGATA
TTGGCGAGGCCAAACCTCCGATCGCACTGGAGGAAAAAGAGTTCACACTGCGGCACATCTA
CCATCATGGCTCCCACGACTACCCAGATCTTCATCGCTATATGGACATTCCAGAGGATGCCA
ATCTGAAGGTCACCAGTGACTTTGGCGCAACATATGAACCAGCTCCTCGTGTCCTCCGCGCC

CGTGCCGCGTCTACCTCCATGCAACGTCTCGCTGACCGTCGTAAAAGTCGTATCGATGGGTT
GTTGGAGCATGCTGAGGTGCATGGGGAGGCAGCCACACTGCCTTCCTCTGCGTGGACTGTGG
ATGAGGTAGCGGGTCCTAACGTAACTGACAAGGAAACCGTGTTGTCATTCGCACGTATGGCA
AATAACGCTTATACTCATGAGCATGGGACGGGGGAGTGGCAAGAAGTCGGCGGCGGGTTTA
ATTATACCGAGGATTTCGGGTGGGAGCAAGACGGTCTCCGTGGTCATATCTTCGCGGATAAA
ACTAACGGTACCATTGTGATTGGGCTGAAGGGTACTTCCATGGCAATTTTTGATGGGGCAGA
AACGACGGGGAATGATAAGTTAAATGACAATCTGTTTGGGTCTTGTTGTTGTGGTCAAGGTG
GGCCTTACGGCTGGAAGCAAGTATGTGACTGCATGACAAATACCTATACTTGTAATTCTACA
TGCTTGGTTAAAAGCCTGCGGCAAAAGTCTCACTATTACTTTGCAGCACGGGATCTGTACCA
CAACGTAACCGAGCGTTACCCAAATAGCGACGTGTGGATGTCGGGTCATTCCCTCGGGGGG
GTAGTAGGCTCTCTGCTTGGTTTAACTTACGGCTTGCCTGTGATGACGTTTGAAGCATATCCA
GATGCTATGGCAGCGTCGCGCTTGGGCCTGCCTACCCCTCCTGGGTACCGTATCGGTGCTCA
CCAATCGCGGCCTTGCACTGGTATCCACCATTATGGTCATACGGCGGACCCTATCTTCATGG
GCACGTGTAACGCGGCGACGAGCCTCTGTACTATTGCCGGGTACGCCCTTCAAAGCCAATGT
CATACGGGGCGTACTTGTATTTACGACACTGTAGGGGACTTAGGTTGGCGCACAGGCGTGGG
TACCCATCGGATCGTGAATGTCATCAAGGATGTAATCGAGAAGTATGACACAGTTCCATCAT
GCGAGGAAGACTTTGAGTGCCAGGACTGTTTCAACTGGAAATTTTTTGAATCTAATTCGTCC
GAAACCACCACATCATCATCCAAACCGTCGTCTACTACGAGCCAGACCCGCACAGAAACCT
GCAAGACCCCTGGGTGGTGGGGGTGTCTCGATGAGACCACCACGCCTCCTACTCCGACAACT
ACAATGACGAGCACGCTGACGACATCATCATGTAAAACCCCAGGCTGGTTTGGCTGCAAGG
ACCCGACGACCATGACGTTGACTACGACCATCACGAGTCCAACGGCGGCTCCTGCACCAATC
GTAACAACTACGTCCAGCACTCCGGTGACTACTACATCGACTTCTTTGGCGTCGCCTTCTTCC
AGTGTAGCCAGTTCCATGAGCACATGTAAGACGCCTGGGTGGTTTGGTTGTAAGGATCCTAC
GTCGACGGTGTCTTCGAACACTGGGTCCTCTACTGCTGCGGCCGCGACGTACAAAGAAGGTT
TGCGCTAGTAATAAGTCGACCGGCTGCTAACAAAGCCCGAAAGGAAGCTGAGTTGGCTGCT
GCCACCGCTGAGCAATAACTAGCATAACCCCTTGGGGCCTCTAAACGGGTCTTGAGGGGTTT
TTTGCTGAAAGCGAGACTAAGCTTTAAACTTCGGGTCATAGCTGTTTCCTG

Needed Genetic Elements by color code:

T7 Promoter

RBS

Start

Protein Sequence –

Stop

T7 Terminator

Circularization site – HindIII Digest

Primer Sequences

**Enzyme 2**

**Cellulase -** *Rachicladosporium antarcticum* **(Fungi)** - Aircraft

# Amino Acids

>TRINITY_DN24997_c0_g2_i7.p1 type:complete len:457 gc:universal
TRINITY_DN24997_c0_g2_i7:1638-268(-)

MVFSNKLTASAASTVALLAALSNA^APAGLMSEVAKRDLNFAFGQEKVRGVNLGGWLVLEPWI
TPSIFEATPDNVVDEYTYGQQYGSDTDRLRDHWNTWITESDFEEMSSIGLNFARLPIGYWSVIGG
EGAPYVNGAYDYVGKAVGWAQNHGIKLMLDLHGAPRSQNGFDNSGQLGNIGWTQGNSVQLT
HQALNKIRDDYASHPSVAAIELVNEPMGSSLDMDTVRQFYMDGWGDLKDSNVAITFHDAFQGV
NSWNDWGSGMWYLLEDTHHYEVFDSGSLQMSASDHVSSACGFGSQMATNNKWTIAGEWSGA
ATDCAQWLNGRGVGARYDGTYNKDGQGSSYIGSCDGKYSGTVEGLSTADKDNLKSFIEAQIVA
FEKAAGWIFWTWKNEAAPEWHFQNLTQQVSKPMGSIAACCCALFAVPSPVAFSAREAVERTAK
RLSLRSHNSFKSRTHANA*

# DNA

GTAAAACGACGGCCAGTAGCGCTATTAAAGCTTCGAAATTAATACGACTCACTATAGGGAG
ACCACAACGGTTTCCCTCTAGAAATAATTTTGTTTAACTTTAAGAAGGAGATATACATATGA
TGGTTTTTAGTAACAAACTGACTGCAAGCGCAGCTTCAACCGTAGCATTATTAGCTGCACTC
AGTAACGCAGCTCCAGCAGGTTTAATGAGCGAAGTTGCCAAACGCGATCTGAACTTCGCGTT
CGGGCAGGAGAAAGTGCGCGGTGTGAACCTTGGCGGCTGGCTCGTCTTAGAACCGTGGATC
ACCCCAAGCATTTTTGAGGCGACTCCAGATAACGTGGTTGACGAGTATACTTACGGTCAACA
ATACGGTAGCGACACCGACCGTCTCCGGGACCACTGGAACACATGGATTACGGAGTCTGAC
TTCGAGGAAATGTCGTCAATCGGCTTGAACTTCGCTCGCCTTCCAATCGGCTATTGGTCCGTC
ATCGGTGGGGAAGGGGCTCCTTATGTAAATGGCGCTTACGACTACGTTGGTAAGGCGGTGG
GCTGGGCGCAGAACCATGGTATTAAGTTGATGTTGGACTTGCACGGCGCACCGCGCAGCCA
AAATGGCTTTGATAATTCGGGCCAATTAGGTAACATTGGGTGGACTCAGGGTAACTCAGTGC
AGTTAACTCATCAAGCGTTGAACAAGATCCGTGACGATTATGCGAGCCACCCATCTGTAGCC
GCAATTGAATTGGTTAATGAACCAATGGGGTCCAGTCTTGATATGGACACCGTTCGCCAATT
TTATATGGACGGCTGGGGGGATTTAAAGGATTCAAATGTAGCAATTACTTTTCACGACGCCT
TTCAAGGGGTCAATTCGTGGAATGATTGGGGCAGTGGGATGTGGTATCTGTTGGAAGATACA
CATCATTACGAGGTTTTTGACAGTGGCAGCCTTCAGATGAGCGCGTCAGATCACGTCAGCTC
CGCGTGCGGGTTCGGCTCACAAATGGCCACTAATAACAAGTGGACTATCGCTGGGGAATGG
TCAGGCGCAGCCACGGATTGTGCACAATGGTTAAACGGCCGGGGGGTAGGCGCGCGGTATG
ACGGGACATATAACAAAGATGGGCAGGGCAGTTCATATATTGGGTCATGTGACGGCAAATA
TAGTGGCACTGTTGAAGGCTTATCGACAGCTGACAAGGATAATTTGAAATCGTTCATCGAAG
CGCAGATTGTTGCCTTTGAAAAAGCGGCCGGTTGGATCTTTTGGACGTGGAAAAATGAAGCA
GCCCCAGAATGGCATTTTCAGAACTTAACGCAACAGGTGAGCAAACCTATGGGGAGCATTG
CCGCGTGTTGCTGCGCGCTTTTCGCTGTTCCGAGTCCTGTTGCATTTTCGGCTCGTGAAGCTG
TTGAGCGGACGGCGAAGCGCCTTTCCCTTCGCAGCCACAATTCGTTTAAGTCGCGGACGCAT
GCTAATGCGTAGTAATAAGTCGACCGGCTGCTAACAAAGCCCGAAAGGAAGCTGAGTTGGC
TGCTGCCACCGCTGAGCAATAACTAGCATAACCCCTTGGGGCCTCTAAACGGGTCTTGAGGG
GTTTTTTGCTGAAAGCGAGACTAAGCTTTAAACTTCGGGTCATAGCTGTTTCCTG

**Enzyme 3**

**Cutinase (#2) -** *Rachicladosporium antarcticum* **(Fungi)** - Aircraft

# Amino Acids

>TRINITY_DN16839_c0_g1_i2.p1 type:complete len:218 gc:universal
TRINITY_DN16839_c0_g1_i2:752-99(-)

MKIPALSCLLFAASTAFAAPSAIEKRTSYNGGLTANDVTDKADCTDLTFIFARGSTERGTMGSTV
GPALAKQLISSLGASKVSVQGVDYEATVSSNALRGSEGGKVMAQLANDQKERCPDTKIAISGYS
QGAMVVHYAVKSAGLDASKVSSAVLYGDPENRESVGDLPSSQTKKYCASGDGVCETGTFSVSA
AHLAYTRNGNIQDGAKFILQQAGLS*

# DNA

GTAAAACGACGGCCAGTAGCGCTATTAAAGCTTCGAAATTAATACGACTCACTATAGGGAG
ACCACAACGGTTTCCCTCTAGAAATAATTTTGTTTAACTTTAAGAAGGAGATATACATATGA
TGAAGATTCCAGCATTAAGCTGTCTCTTATTTGCGGCTTCCACCGCCTTCGCCGCTCCATCGG
CTATCGAGAAGCGCACCTCGTATAATGGGGGCTTGACAGCAAATGATGTGACTGATAAGGC
TGATTGCACTGACCTCACATTCATCTTCGCTCGCGGTTCCACAGAACGGGGGACGATGGGGA
GTACGGTAGGGCCTGCGCTGGCTAAACAACTTATCTCAAGTCTTGGCGCTTCCAAGGTTTCG
GTTCAAGGGGTAGACTATGAGGCGACGGTGAGCTCTAACGCCCTCCGTGGGAGCGAGGGGG
GCAAAGTGATGGCCCAATTAGCTAATGACCAGAAGGAACGTTGTCCAGACACCAAAATTGC
CATCTCCGGTTACTCGCAAGGGGCGATGGTGGTCCACTATGCGGTAAAATCAGCAGGCTTAG
ACGCGTCTAAGGTGAGTAGTGCCGTGCTCTACGGGGATCCAGAAAACCGGGAAAGCGTCGG
TGACTTACCTTCTAGCCAAACGAAGAAATACTGTGCGTCTGGTGACGGCGTTTGTGAGACGG
GTACATTCTCTGTAAGTGCTGCTCATCTCGCTTATACGCGCAACGGTAACATTCAGGACGGC
GCAAAATTCATTCTTCAACAAGCAGGTTTATCCTGATAATAAGTCGACCGGCTGCTAACAAA
GCCCGAAAGGAAGCTGAGTTGGCTGCTGCCACCGCTGAGCAATAACTAGCATAACCCCTTG
GGGCCTCTAAACGGGTCTTGAGGGGTTTTTTGCTGAAAGCGAGACTAAGCTTTAAACTTCGG
GTCATAGCTGTTTCCTG

**Enzyme 4**

**Cutinase (#3) -** *Hortaea werneckii* **(Fungi)** - Aircraft

# Amino Acids

>TRINITY_DN25911_c0_g1_i3.p1 type:complete len:439 gc:universal
TRINITY_DN25911_c0_g1_i3:1417-101(-)

MSTIIALSASLLLAAPAALAQSTGTGSCTDVHIFLAKGWNEQYNDQRQTKLVDAICSDLGSNVSC
DYEDIILNDLAGSDYCTAVTEGTGNGKQQITAYAAKCPNSKLVLSGYSEGADVVGDIMAGGGG
TYDGHCATTSPLDASSDAVCQLAAVMVFGNPRHVPNTSYNVDSGVAGLGQYPRTPEQAAILAS
YADKLHDWCNINDPTCANGLGTNQAEPWHTNYFDLVTDEAASWAVSKIDAAKSCAKMTSSSSS
SSMMASTSTKAAMATSSSAAMMSSSSAMMASSSSTMMPSSSSAMMSGPAGYGMSSSMMASAS
MTKPASYPTTTAAANMTSYPSSVTTMISGKPACVSYAMTSSMTTWGPYPAYPVSSSYPVTTSWI
GWGNGTAATMTMMGSASGTGAAKATGSGMPSYTGAASGVKAAGGLAVAAVVGVVALMA*

# DNA

GTAAAACGACGGCCAGTAGCGCTATTAAAGCTTCGAAATTAATACGACTCACTATAGGGAG
ACCACAACGGTTTCCCTCTAGAAATAATTTTGTTTAACTTTAAGAAGGAGATATACATATGA
TGAGTACGATTATCGCCCTTAGTGCCTCCTTGTTGCTTGCAGCGCCTGCCGCCTTGGCCCAGA
GCACTGGGACCGGCTCCTGTACCGATGTTCACATTTTTCTCGCAAAAGGTTGGAATGAGCAG
TATAATGACCAACGCCAAACAAAACTCGTAGATGCCATCTGTTCCGACCTGGGGTCTAATGT
TTCATGCGACTATGAAGACATTATCCTTAATGATTTAGCCGGTTCTGATTATTGCACAGCAGT
AACTGAAGGTACAGGGAATGGCAAACAGCAGATCACTGCATACGCAGCGAAGTGTCCTAAT
TCTAAGTTAGTTCTTTCTGGGTATTCTGAGGGTGCAGACGTCGTTGGCGATATTATGGCTGGT
GGCGGTGGCACTTACGACGGCCACTGTGCAACGACCAGCCCGCTGGATGCGAGCTCGGATG
CTGTATGTCAACTTGCTGCGGTTATGGTGTTCGGGAATCCGCGCCATGTTCCTAATACATCCT
ATAACGTGGATTCTGGTGTTGCGGGCCTTGGGCAATACCCACGTACCCCAGAGCAAGCCGCT
ATTCTTGCTAGTTACGCGGACAAGCTCCATGATTGGTGCAACATTAATGATCCAACGTGTGC
GAACGGCCTTGGCACTAACCAAGCGGAGCCGTGGCACACAAATTACTTTGACCTCGTTACGG
ATGAAGCAGCTAGTTGGGCTGTTTCCAAAATTGACGCTGCTAAGTCCTGCGCCAAAATGACC
TCATCCTCGAGCTCCAGTTCTATGATGGCATCCACATCGACTAAAGCAGCTATGGCCACTAG
TTCGTCCGCGGCAATGATGAGTAGCAGTTCTGCGATGATGGCCTCTTCGTCCTCCACAATGA
TGCCTTCTTCGTCTAGTGCGATGATGAGCGGTCCGGCTGGGTACGGATGTCTTCAAGTATG
ATGGCATCAGCATCAATGACGAAGCCGGCGAGCTACCCAACCACGACGGCTGCTGCCAACA
TGACATCGTATCCGTCGAGTGTCACTACCATGATCAGTGGCAAGCCAGCATGTGTATCTTAC
GCTATGACAAGTTCTATGACTACGTGGGGCCCATACCCAGCCTATCCGGTAAGTTCTTCTTAC
CCGGTGACAACCAGCTGGATTGGCTGGGGCAATGGGACCGCGGCGACGATGACTATGATGG
GTTCAGCTTCTGGCACCGGGGCAGCGAAGGCCACGGGGAGTGGGATGCCTTCGTATACCGG
TGCCGCTTCGGGTGTAAAAGCGGCCGGGGGTCTTGCAGTAGCGGCGGTGGTTGGCGTGGTCG
CCCTTATGGCATAGTAATAAGTCGACCGGCTGCTAACAAAGCCCGAAAGGAAGCTGAGTTG
GCTGCTGCCACCGCTGAGCAATAACTAGCATAACCCCTTGGGGCCTCTAAACGGGTCTTGAG
GGGTTTTTTGCTGAAAGCGAGACTAAGCTTTAAACTTCGGGTCATAGCTGTTTCCTG

**Enzyme 5**

**Cutinase (#4) -** *Hortaea werneckii* **(Fungi) -** Aircraft

# Amino Acids

>TRINITY_DN18896_c0_g3_i1.p1 type:complete len:225 gc:universal
TRINITY_DN18896_c0_g3_i1:466-1140(+)

MKFTAAIALLASTAAALPTAVERRQFGTRVGSTVNELTNGACRPITFIFARGSTEIGNIGSTVGPPT
CEGLKSEYGANQVACQGVGSPYQATIGANALPEGTTSAAYGEAQRLFNLASTKCPDTIIVAGGY
SQGAAVMTAAVRRLSSSVQDKIAGVVLYGNTRNAQNNGKIPNFPPEKALTFCNLTDGVCGGGL
VVTAGHLTYTRDVDDAVDYLNERITAAGGI*

# DNA

GTAAAACGACGGCCAGTAGCGCTATTAAAGCTTCGAAATTAATACGACTCACTATAGGGAG
ACCACAACGGTTTCCCTCTAGAAATAATTTTGTTTAACTTTAAGAAGGAGATATACATATGA
TGAAGTTCACGGCTGCAATCGCATTATTAGCGTCGACCGCTGCCGCCTTGCCAACCGCAGTA
GAACGGCGGCAGTTTGGGACACGGGTAGGCTCTACGGTGAATGAATTGACGAATGGTGCCT

GTCGGCCGATTACATTTATTTTTGCTCGTGGCTCCACCGAGATCGGCAATATTGGTTCAACCG
TAGGTCCGCCAACATGCGAGGGCCTGAAGAGCGAGTATGGCGCCAACCAAGTGGCTTGCCA
GGGGGTTGGCTCCCCATATCAAGCCACCATTGGGGCAAATGCACTGCCTGAGGGCACGACA
TCGGCAGCATATGGCGAGGCACAGCGTTTATTCAACTTAGCAAGCACGAAATGTCCAGACA
CAATCATCGTAGCGGGCGGCTACAGCCAAGGGGCGGCAGTCATGACCGCAGCGGTACGTCG
TTTGTCCTCCAGCGTGCAGGACAAGATCGCTGGGGTCGTTCTGTACGGGAACACACGGAACG
CTCAGAACAATGGGAAAATCCCAAACTTCCCTCCTGAGAAAGCCCTTACCTTCTGCAATCTC
ACCGATGGGGTGTGTGGCGGTGGGTTGGTAGTTACAGCTGGTCATCTGACCTATACACGCGA
TGTCGATGACGCTGTCGATTACTTAAACGAACGGATCACAGCTGCGGGGGGTATTTAGTAAT
AAGTCGACCGGCTGCTAACAAAGCCCGAAAGGAAGCTGAGTTGGCTGCTGCCACCGCTGAG
CAATAACTAGCATAACCCCTTGGGGCCTCTAAACGGGTCTTGAGGGGTTTTTTGCTGAAAGC
GAGACTAAGCTTTAAACTTCGGGTCATAGCTGTTTCCTG

**Enzyme 6**

**Cutinase (#5) -** *Rachicladosporium antarcticum* **(Fungi) -** Aircraft

# Amino Acids

>TRINITY_DN24694_c0_g1_i2.p1 type:complete len:229 gc:universal
TRINITY_DN24694_c0_g1_i2:663-1349(+)

MLSKTALLALVASVAASPIDLSERASCPQIHVFGARETTASPGLGSAATVVNGILSAHSGATSEAI
DYPACGGGSSCGGVSYPNSVKQGTAAVISQVSAFAKQCPNTEIVMVGYSQGAEIIDTVLCGNGD
PKQSVQSTGSIAGYNIKAAIFMGDPRYETGAPYNVGTCKAGGFSSRPAGQTCGNYNSKIQSYCD
AADPYCCNGSNAATHNGYGQEYGQQAIAFVNGKL*

# DNA

GTAAAACGACGGCCAGTAGCGCTATTAAAGCTTCGAAATTAATACGACTCACTATAGGGAG
ACCACAACGGTTTCCCTCTAGAAATAATTTTGTTTAACTTTAAGAAGGAGATATACATATGA
TGTTGTCGAAGACGGCACTGTTGGCTCTTGTGGCCTCAGTTGCTGCCAGTCCGATTGACCTTT
CTGAGCGTGCCTCTTGTCCTCAGATCCATGTATTCGGGGCTCGGGAGACGACAGCGAGTCCT
GGTCTTGGCTCAGCGGCGACAGTGGTGAACGGGATCCTTTCTGCACACTCGGGGGCTACCAG
CGAAGCTATTGATTACCCAGCTTGCGGCGGCGGCAGTTCATGTGGGGGCGTCAGTTACCCTA
ATTCTGTGAAACAGGGCACCGCCGCGGTGATTTCGCAGGTCTCTGCGTTTGCAAAACAATGC
CCAAATACTGAGATTGTAATGGTCGGTTACTCGCAGGGCGCTGAGATCATCGATACAGTTTT
GTGCGGGAATGGGGATCCAAAACAATCAGTACAGTCAACAGGGAGTATCGCCGGGTATAAC
ATCAAGGCCGCAATCTTTATGGGGGATCCACGTTATGAAACAGGCGCGCCGTACAATGTCGG
CACCTGTAAGGCGGGTGGTTTTTCGAGCCGTCCGGCTGGGCAAACTTGCGGTAATTACAACA
GTAAAATCCAGAGCTACTGTGATGCGGCCGACCCATATTGCTGTAATGGTTCTAATGCAGCC
ACTCATAACGGCTATGGTCAGGAGTACGGGCAACAGGCTATCGCATTTGTTAATGGGAAACT
TTAATAATAAGTCGACCGGCTGCTAACAAAGCCCGAAAGGAAGCTGAGTTGGCTGCTGCCA
CCGCTGAGCAATAACTAGCATAACCCCTTGGGGCCTCTAAACGGGTCTTGAGGGGTTTTTTG
CTGAAAGCGAGACTAAGCTTTAAACTTCGGGTCATAGCTGTTTCCTG

**Enzyme 7**

**COesterase (#1) -** *Peltaster fructicola* **(Fungi) -** Aircraft

# Amino Acids

>TRINITY_DN22849_c0_g1_i2.p1 type:complete len:570 gc:universal
TRINITY_DN22849_c0_g1_i2:1766-57(-)

MLTILASLLAVASAAPPSYGSPPSYGSGPSVHLKNGTVAGSHNSAYNQDFFLGVPYAQPPVNGLR
FRNPQSLNSTYNSTLQATAYAPSCVGYGGDDIGYPVSEDCLYLNVVRPAGHEGKDLPVGFWIHG
GGLVMGGSRDERYNLSFIVENGVKIGKPFIGVSINYRLAGWGFLASQEVSGAGQTNIGLRDQRLA
LHWIQENIGAFGGDPSKVTIWGESAGAASVGWHLTAYNGRDDKLFRGGIMESGNPVNYNSYRT
ETHYQPGYDDLVNRTGCANATDTLDCLRYAPYDTVNNFFNSTAGSDFSPIVDGDFIQRWASIQL
AEGDFVKVPIIDGANTDEGTSFGPVGISTDAEFVAYASNSSAAQAFLPATIAPQVLEAYPDIPSYFI
PPVAEIGNYTYNASTYGAQYRRTAAYGGDVVMIANRRGACQTWAANGLKAYSYRFNTRPNGL
PPSVGVTHFQEVAFVFDNTQGLGYNAAHGTVNPFGNKPQSYKDLAYLMSSSWASFIASLDPND
WEGRDKATPAWPAYDNANPQNIVWDANVTALAFAEPDTWRKEGIQFILDHAKAYHR*

# DNA

GTAAAACGACGGCCAGTAGCGCTATTAAAGCTTCGAAATTAATACGACTCACTATAGGGAG
ACCACAACGGTTTCCCTCTAGAAATAATTTTGTTTAACTTTAAGAAGGAGATATACATATGA
TGTTGACTATTCTCGCATCACTGTTGGCTGTTGCGAGCGCGGCGCCGCCAAGTTATGGGTCA
CCGCCTAGTTACGGGTCGGGCCCTTCCGTTCACTTGAAAAACGGTACGGTCGCAGGGTCACA
CAACTCTGCATACAACCAAGATTTTTTCCTCGGTGTGCCGTATGCCCAGCCACCGGTGAACG
GCTTACGTTTTCGGAACCCACAATCACTCAACTCCACATATAATTCGACATTGCAGGCCACT
GCCTATGCACCGTCGTGCGTCGGGTATGGTGGTGACGATATTGGTTATCCGGTATCTGAAGA
CTGCCTTTACCTTAATGTAGTACGCCCAGCGGGGCACGAAGGGAAAGACCTGCCAGTAGGG
TTCTGGATTCATGGCGGGGGGCTTGTGATGGGTGGTAGCCGGGATGAACGTTATAACTTGTC
TTTCATCGTTGAGAACGGGGTTAAGATTGGCAAACCATTTATCGGTGTCTCCATCAACTACC
GCCTGGCAGGTTGGGGTTTTCTCGCCTCGCAAGAGGTCAGTGGCGCCGGCCAGACGAACATC
GGGCTGCGGGATCAACGTCTCGCATTACACTGGATTCAGGAGAACATCGGGGCCTTTGGTGG
GGACCCTAGTAAAGTAACCATCTGGGGCGAATCGGCTGGCGCGGCTAGCGTGGGTTGGCAC
CTGACAGCATACAACGGCCGGGACGATAAGCTGTTTCGTGGGGGTATTATGGAATCAGGTA
ACCCAGTGAATTACAATAGCTATCGTACAGAAACACATTACCAACCTGGCTACGACGACTTG
GTTAACCGGACGGGGTGCGCAAACGCAACTGATACGTTAGACTGTTTACGCTATGCGCCGTA
TGACACTGTGAATAATTTCTTTAACAGCACAGCCGGCTCGGACTTCAGTCCGATCGTGGACG
GGGACTTTATTCAGCGGTGGGCATCGATTCAGTTAGCAGAGGGTGATTTCGTGAAGGTACCG
ATTATCGACGGGGCGAATACAGATGAAGGCACTAGCTTCGGTCCGGTCGGTATCAGTACGG
ATGCCGAGTTCGTAGCTTATGCCAGTAACAGCTCAGCGGCCCAAGCGTTTCTCCCGGCCACC
ATCGCGCCGCAAGTTCTGGAAGCCTACCCGGATATTCCATCCTACTTCATTCCTCCAGTAGCA
GAGATTGGCAACTACACCTACAACGCCTCAACTTACGGCGCTCAGTACCGGCGTACAGCTGC
GTACGGTGGGGATGTAGTTATGATTGCAAACCGGCGTGGTGCTTGTCAAACCTGGGCTGCAA
ATGGCTTGAAGGCCTATTCATACCGGTTCAATACTCGCCCAAACGGGTTGCCGCCGTCCGTG
GGTGTCACCCACTTTCAAGAAGTCGCTTTCGTATTTGATAACACACAGGGCCTTGGGTACAA
TGCCGCGCATGGCACGGTAAACCCTTTCGGGAATAAACCACAAAGTTATAAGGACCTGGCA
TACCTGATGAGTTCTTCATGGGCGTCTTTTATTGCCAGCCTTGATCCAAACGACTGGGAGGG
CCGGGATAAGGCAACACCTGCTTGGCCGGCCTATGACAACGCGAATCCGCAAAACATTGTG

TGGGATGCAAACGTGACAGCACTCGCTTTTGCCGAGCCGGACACCTGGCGGAAGGAGGGCA
TCCAATTTATCTTGGACCATGCAAAGGCCTACCACCGTTAGTAATAAGTCGACCGGCTGCTA
ACAAAGCCCGAAAGGAAGCTGAGTTGGCTGCTGCCACCGCTGAGCAATAACTAGCATAACC
CCTTGGGGCCTCTAAACGGGTCTTGAGGGGTTTTTTGCTGAAAGCGAGACTAAGCTTTAAAC
TTCGGGTCATAGCTGTTTCCTG

**Enzyme 8**

**COesterase (#2) -** *Hortaea werneckii* **(Fungi)** - Aircraft

# Amino Acids

>TRINITY_DN25137_c0_g2_i2.p2 type:complete len:527 gc:universal
TRINITY_DN25137_c0_g2_i2:2706-4286(+)

MGYTTLLGALALAPALSLAAPTASQLTVRASTGYYTGQINSNFTNVREFLDVPYGVTTSGANRF
MPPIAVPLSSKHYNSTAYPPACPQYVTAVKNIWNQQIPQYLQYWGNSNNSAGESAVFTSEDCLK
LAIWTPANATSASNLPVALFWTGGGFQTNGILVPGQLPPGWVEKSQSHIVVTINYRMNIMGFPN
AAGVSEQNLGLMDQRVSLEWVRDNIRYFGGDPSKIMIWGQSAGASSVDYHNYAYWDEPIAHAI
FAESGSAYPGTAWRDNSNFTFVASNLGCSYPNNATQELQCMQKVDYNKIINFMGQYQDNSTLH
PSTPQPPLTFSAVADERLVFANYTQRYLEGFVSKVPMIYSSVANEGGSLQPYPINNPYNGTNQSL
ANTITEGVLCGASNSTILRHSIGLPTFRYQYAGNWTNQDPLPWMGAFHSSDLVMLMGSYRTGGG
PPKEALEGETSDTMGEYVLAFMRDPWNGPQKLGWYPMDPSEADGGSMLRFGADGRAVQNVT
GYEVQKVCFGEGSYDPFP*

# DNA

GTAAAACGACGGCCAGTAGCGCTATTAAAGCTTCGAAATTAATACGACTCACTATAGGGAG
ACCACAACGGTTTCCCTCTAGAAATAATTTTGTTTAACTTTAAGAAGGAGATATACATATGA
TGGGCTATACCACTTTGCTCGGCGCTTTGGCCCTGGCACCAGCTTTATCCCTGGCTGCTCCTA
CGGCATCTCAGTTAACCGTACGTGCAAGCACTGGCTACTATACCGGCCAAATTAACAGTAAT
TTTACAAACGTGCGGGAGTTCCTTGATGTTCCATACGGTGTCACGACGAGCGGCGCGAATCG
GTTTATGCCTCCGATCGCGGTACCACTGTCCAGCAAGCACTATAACTCAACAGCATATCCTC
CAGCCTGTCCTCAGTATGTCACTGCAGTCAAAAACATTTGGAACCAACAGATTCCGCAATAC
TTGCAATACTGGGGTAACTCTAACAACTCGGCAGGCGAAAGCGCTGTTTTCACATCGGAAGA
TTGTTTAAAATTGGCGATTTGGACCCCTGCTAATGCCACCAGTGCAAGCAATCTCCCAGTGG
CTCTGTTTTGGACTGGTGGTGGCTTTCAGACCAACGGCATCTTGGTTCCGGGCCAGCTCCCAC
CAGGGTGGGTGGAGAAGTCCCAGAGTCATATTGTCGTTACCATCAATTACCGTATGAACATT
ATGGGTTTCCCAAATGCGGCGGGCGTATCAGAACAAAACTTGGGCCTGATGGATCAGCGGG
TCTCCCTTGAGTGGGTACGTGATAATATTCGGTATTTCGGCGGTGATCCATCTAAAATCATGA
TTTGGGGTCAATCCGCTGGGGCAAGCTCTGTCGATTACCATAATTACGCTTACTGGGACGAG
CCTATCGCCCATGCTATTTTCGCGGAATCTGGTTCTGCGTACCCAGGTACAGCCTGGCGGGA
CAATTCTAATTTCACATTCGTTGCATCTAATCTGGGCTGTTCTTACCCGAACAACGCCACCCA
AGAGCTGCAATGTATGCAAAAGGTTGATTATAATAAGATTATTAACTTTATGGGCCAGTATC
AGGACAATAGTACTTTGCACCCAAGCACGCCACAACCACCTCTTACTTTTAGCGCTGTGGCA
GATGAGCGGCTTGTGTTCGCGAACTATACTCAACGCTACTTGGAGGGTTTCGTGTCCAAGGT
TCCGATGATCTACTCGAGTGTTGCCAACGAAGGCGGCTCCCTGCAGCCATATCCAATCAACA
ACCCGTACAACGGTACAAACCAGAGCTTAGCTAATACGATTACCGAGGGCGTTCTTTGCGGG
GCAAGCAATTCCACAATTCTGCGCCACAGCATCGGCCTGCCTACCTTCCGCTATCAGTACGC
GGGCAATTGGACAAACCAGGATCCATTACCGTGGATGGGCGCGTTCCATTCATCTGACTTAG

TAATGTTAATGGGCTCGTACCGGACAGGCGGCGGTCCACCAAAAGAGGCGCTGGAGGGGGA
AACATCTGACACCATGGGGGAGTATGTCTTGGCATTTATGCGCGATCCTTGGAACGGGCCTC
AGAAGTTAGGCTGGTACCCAATGGATCCGTCAGAGGCGGATGGCGGTTCTATGTTACGCTTC
GGGGCCGACGGTCGCGCGGTTCAGAACGTGACGGGCTACGAAGTTCAGAAGGTATGTTTTG
GGGAAGGTAGTTACGATCCTTTTCCATGATAATAAGTCGACCGGCTGCTAACAAAGCCCGAA
AGGAAGCTGAGTTGGCTGCTGCCACCGCTGAGCAATAACTAGCATAACCCCTTGGGGCCTCT
AAACGGGTCTTGAGGGGTTTTTTGCTGAAAGCGAGACTAAGCTTTAAACTTCGGGTCATAGC
TGTTTCCTG

**Enzyme 9**

**Peptidase_S28 (#1) -** *Friedmanniomyces simplex* **(Fungi) -** Aircraft

# Amino Acids

>TRINITY_DN25166_c0_g2_i3.p1 type:complete len:524 gc:universal
TRINITY_DN25166_c0_g2_i3:2430-859(-)

MRSLYNYLSIALCLTISSSPAAVPSTKHEQEPLSTPKHLRAIAATVQLPIDHFNASDTRTFANRFW
YNDTFYRPGGPVLFYDEGERGIADGVVPPAFYPDHPVTLLAEKFGALVVAWEHRFYGLSTPFPD
LNVTSPHEEMERAFAYLDTEQALEDVVVFARSFELPGLEKALQPNATPWIWIGGSYAGQRAVMI
RKRNPGTFWASWSSSAPIEVALEFPEYYLEVSRDLPRKCREVIRRAIERVDEVLLRGSGMRRLRL
RWEIVRRWDEGRSWREKMQFVGFAPDFVVAEWFMRVVAVDWQAEGMKGGMNATCRRLTLH
DDCGADDPDADITAVLDAIASSENSSGRPPSSFFNFPMDELAWRYQISTEYPYLQTSKPSSPYNILS
SFLDFNSTWHYHHELSFPCITSPPNVSALIPTRYAGWDVGLAKRVMITSGLRDPWHQLSALPKDS
LVPGAPVNRSARAAVPGCHEDLAENEVFGLVLERGRHCADLVIGNPEAKQATALFAQALEVWL
PCFVNDTSR*

# DNA

GTAAAACGACGGCCAGTAGCGCTATTAAAGCTTCGAAATTAATACGACTCACTATAGGGAG
ACCACAACGGTTTCCCTCTAGAAATAATTTTGTTTAACTTTAAGAAGGAGATATACATATGA
TGCGCAGTTTATACAACTATCTGAGCATCGCTTTGTGCTTAACTATCAGCAGCAGTCCTGCAG
CAGTACCATCCACAAAGCACGAACAGGAGCCACTGTCGACGCCAAAACACCTGCGCGCCAT
TGCGGCTACAGTTCAGTTGCCAATCGACCATTTCAATGCGAGCGATACTCGGACTTTCGCAA
ACCGCTTTTGGTATAATGATACCTTCTATCGGCCGGGTGGTCCAGTGCTTTTTTACGACGAAG
GGGAACGCGGGATCGCTGATGGCGTTGTGCCACCAGCATTTTACCCAGATCATCCTGTGACG
CTTTTAGCCGAGAAGTTCGGTGCGCTTGTTGTAGCGTGGGAGCACCGCTTCTACGGCCTGTC
AACGCCTTTCCCAGACTTGAACGTCACGAGCCCGCATGAGGAGATGGAGCGCGCATTTGCCT
ATTTGGACACTGAACAGGCCCTGGAAGACGTCGTCGTATTTGCCCGTTCATTCGAATTACCG
GGTTTAGAAAAGGCACTTCAACCTAATGCCACACCTTGGATCTGGATTGGGGGCTCTTACGC
CGGCCAACGTGCCGTCATGATCCGCAAACGCAATCCTGGTACGTTCTGGGCTTCTTGGTCTTC
GAGCGCCCCAATCGAAGTCGCCTTAGAGTTCCCTGAGTACTATCTCGAGGTTTCGCGGGATC
TTCCGCGCAAGTGCCGCGAAGTGATCCGGCGTGCGATTGAACGCGTCGATGAGGTGCTCTTG
CGGGGCTCTGGGATGCGTCGCTTGCGGTTACGTTGGGAAATTGTACGCCGGTGGGATGAAGG
CCGGAGCTGGCGTGAGAAGATGCAGTTCGTCGGGTTTGCTCCGGATTTCGTAGTAGCAGAAT
GGTTCATGCGCGTTGTAGCTGTTGATTGGCAAGCGGAAGGCATGAAGGGTGGCATGAATGC
GACGTGCCGGCGTCTCACGCTGCACGATGATTGTGGTGCTGATGATCCTGACGCGGACATCA

CAGCAGTTCTCGACGCAATCGCCTCGAGCGAGAATTCTTCGGGTCGTCCACCTTCATCTTTCT
TCAATTTTCCGATGGACGAACTTGCCTGGCGCTACCAGATTTCCACAGAATACCCGTACCTTC
AGACCAGTAAGCCTTCTTCTCCATATAATATTCTGAGCTCGTTTTAGACTTTAACTCCACGT
GGCATTACCATCATGAACTTTCCTTTCCGTGTATCACAAGTCCTCCAAACGTAAGCGCCTTGA
TTCCTACCCGGTATGCTGGCTGGGATGTCGGCCTTGCCAAACGGGTAATGATCACGTCCGGG
CTTCGGGATCCGTGGCACCAACTTTCGGCGCTTCCGAAAGATTCCTTAGTACCTGGCGCACC
AGTAAACCGGAGCGCCCGCGCGGCAGTTCCGGGTTGTCACGAAGACCTCGCAGAAAATGAG
GTCTTTGGTCTGGTCTTGGAACGGGGTCGCCACTGCGCGGATCTTGTTATTGGCAACCCAGA
AGCGAAGCAAGCCACTGCCCTTTTCGCACAGGCACTGGAGGTATGGCTTCCATGCTTTGTAA
ATGATACCAGTCGCTAGTAATAAGTCGACCGGCTGCTAACAAAGCCCGAAAGGAAGCTGAG
TTGGCTGCTGCCACCGCTGAGCAATAACTAGCATAACCCCTTGGGGCCTCTAAACGGGTCTT
GAGGGGTTTTTTGCTGAAAGCGAGACTAAGCTTTAAACTTCGGGTCATAGCTGTTTCCTG

**Enzyme 10**

**Lipase_3 (#2) -** *Coccomyxa subellipsoidea* **C-169 (Fungi) -** Aircraft

# Amino acids

>TRINITY_DN40427_c2_g1_i1.p1 type:complete len:657 gc:universal
TRINITY_DN40427_c2_g1_i1:2484-514(-)

MRSALYLLALFVLSRPGACQLGGFIDNIGNSISNALGGGSGSAASPAQSADAALGGTFVQLRNVN
PYIAGPIWFKNADAAQYAFVAQNNMSLDGSLPNARRIPLASGCVAVVSYDTSNETATIAFAGPA
DVQNRAASTTSTSNLQQVDFLSLFIPAAQAIPEVLAIFDSAIGAGNSSSLSEEIDFLSGGAVPMRVV
CTGYGIGGSLADLCGVWAGRLYPDTKVRVMTFGAPFVGNDAYAYAHQQLVDLSYLWVLTSGP
SQISTLLPHSVTNNSMAQSGSTAGTLTNYTNIILQRSFQTSSVPALSIEPTPTVIPNSSISDWFSIVDG
LVAPDGSASSSTSAASFDDIVVPSIPINDSDIREAFQFTDPTTPDPSCPAVLCKMQGEAAAACGVY
QIGNDSSVTETTNAIPGSVLVTGTSSGAHVAVAYNSSTKIALISWRGSVSDRSWIDDAELVQVDY
QWPGQTFTKTGLYNLADTFPGPPQVHTGFYGELQDVSTSAKSDATNITKILQNMIGSDTPIRIVLT
GHSLGAAVSSINAFQYAINWPMADIHNVNLGSPLVGDQNWVNAFRGLVGRAYRGVNAHDQVP
ALPPLSQFRHVGYGVWIDNGVPKLQDRPYLDVMDTTWDNHTCSNYTGYLYNATQVYIPTFPLS
VNNAVDM*

# DNA

GTAAAACGACGGCCAGTAGCGCTATTAAAGCTTCGAAATTAATACGACTCACTATAGGGAG
ACCACAACGGTTTCCCTCTAGAAATAATTTTGTTTAACTTTAAGAAGGAGATATACATATGA
TGCGGAGCGCGCTGTATCTCTTAGCGCTGTTCGTGCTGTCCCGTCCAGGTGCTTGTCAACTTG
GGGGCTTCATTGATAATATCGGGAACTCTATCTCCAACGCCTTAGGGGGTGGTTCAGGTTCA
GCGGCCTCGCCTGCACAGTCAGCCGACGCAGCATTAGGTGGGACATTTGTACAACTTCGCAA
CGTGAATCCGTATATTGCAGGGCCAATTTGGTTCAAAAACGCTGACGCCGCGCAATATGCAT
TTGTTGCTCAGAATAATATGTCCTTGGACGGTTCATTGCCGAATGCTCGCCGTATTCCACTTG
CATCTGGCTGTGTGGCAGTGGTGTCGTACGATACTTCTAATGAGACTGCGACCATCGCCTTT
GCGGGTCCTGCTGACGTACAAAACCGCGCCGCATCCACAACTTCCACTTCGAATCTCCAGCA
AGTGGACTTTTTGTCCCTCTTTATTCCTGCAGCCCAGGCAATTCCGGAAGTACTGGCAATTTT
TGATTCGGCAATCGGGGCTGGCAACTCAAGCAGCCTGTCTGAAGAGATTGACTTTTTGTCTG
GTGGCGCTGTGCCAATGCGCGTCGTGTGCACAGGCTACGGTATTGGTGGGAGTCTTGCAGAT

TTATGCGGTGTGTGGGCGGGTCGTCTTTATCCTGATACAAAGGTACGTGTTATGACTTTCGGT
GCACCATTTGTGGGCAATGACGCGTACGCGTATGCCCATCAACAGTTGGTGGACCTTTCTTA
TTTGTGGGTACTCACCTCCGGCCCGTCGCAGATTTCAACACTCCTCCCACATTCCGTGACTAA
TAATTCAATGGCTCAGTCAGGGTCAACGGCTGGGACCCTGACGAATTATACGAATATTATCC
TTCAGCGCTCATTCCAAACGTCGTCAGTACCTGCTCTGAGCATTGAACCGACTCCTACAGTTA
TTCCTAATTCTTCCATCTCGGACTGGTTCTCTATCGTAGATGGGCTTGTCGCCCCTGACGGTA
GCGCAAGCTCAAGCACATCGGCTGCTTCCTTTGATGATATCGTCGTACCATCCATCCCTATCA
ACGATAGCGACATTCGGGAAGCCTTCCAGTTCACGGACCCGACGACGCCTGACCCTTCATGT
CCGGCTGTGTTATGTAAGATGCAAGGTGAGGCGGCAGCGGCGTGCGGGGTATACCAGATCG
GTAATGACTCTAGCGTCACTGAAACTACTAACGCTATTCCTGGTTCGGTGTTAGTTACGGGT
ACGTCGAGTGGGGCACACGTAGCTGTTGCTTACAACAGCTCGACAAAAATCGCCCTGATCTC
CTGGCGGGGGTCGGTCAGCGACCGCTCATGGATCGACGATGCGGAACTGGTACAGGTAGAC
TATCAGTGGCCGGGCCAAACTTTCACAAAGACGGGTTTGTACAATTTAGCAGACACCTTCCC
AGGGCCACCACAGGTACACACCGGTTTTTACGGGGAATTGCAGGATGTATCTACCTCCGCCA
AGTCGGATGCGACCAACATCACAAAGATTTTGCAGAATATGATCGGGAGTGACACCCCGAT
TCGCATCGTATTAACCGGGCATTCACTCGGCGCCGCCGTGAGTAGTATTAATGCGTTTCAAT
ATGCTATCAATTGGCCTATGGCTGATATTCACAATGTCAACCTCGGTTCTCCTCTTGTCGGGG
ATCAAAATTGGGTAAATGCTTTTCGGGGCCTCGTCGGCCGCGCGTACCGTGGGGTTAACGCG
CACGATCAGGTGCCTGCGCTCCCTCCATTATCGCAATTTCGCCACGTTGGTTACGGTGTTTGG
ATTGATAATGGTGTGCCAAAGCTCCAGGACCGTCCTTACTTGGACGTGATGGACACGACCTG
GGATAACCACACTTGCAGCAATTACACAGGCTACTTGTATAATGCGACCCAGGTCTATATTC
CGACCTTCCCACTTTCTGTAAATAATGCAGTCGATATGTGATAATAAGTCGACCGGCTGCTA
ACAAAGCCCGAAAGGAAGCTGAGTTGGCTGCTGCCACCGCTGAGCAATAACTAGCATAACC
CCTTGGGGCCTCTAAACGGGTCTTGAGGGGTTTTTTGCTGAAAGCGAGACTAAGCTTTAAAC
TTCGGGTCATAGCTGTTTCCTG

**Enzyme 11**

**Dienelactone hydrolase - *Coleofasciculus sp.* (Bacteria-)** - Truck

# Amino Acids

>TRINITY_DN38084_c1_g1_i1.p1

MSVTRRKFIIVSTLAAGFAACTHPISAQVVTTDTQGLIAGEVKIPVADGEIPAYRAMPATGENFPV
VLVVQEIFGVHEHIQDVCRRFAKLGFVAIASELFARQGDPTQLGNSQEIISKIVSKVPDAQVMSDL
DATVAWAGKSSKGNLNKLAITGFCWGGRIVWLYAAHNPQVKAGVAWYGRLVGDVTPLTPKH
PVDIASELKTPILGLYGGSDDGIPVATVEQMRDRLKAGRSGSEIIVYPDTPHAFFADYRPSYRKEQ
AEDGWKRLQAWFDVKISLADTLLFF*

# DNA

GTAAAACGACGGCCAGTAGCGCTATTAAAGCTTCGAAATTAATACGACTCACTATAGGGAG
ACCACAACGGTTTCCCTCTAGAAATAATTTTGTTTAACTTTAAGAAGGAGATATACATATGA
TGTCCGTAACCCGGCGTAAATTCATTATCGTCTCTACTCTGGCGGCAGGCTTCGCCGCCTGCA
CCCATCCAATTAGCGCCCAGGTAGTCACGACAGATACTCAAGGCCTGATCGCCGGGGAGGT
AAAAATTCCTGTAGCAGACGGCGAGATCCCAGCGTATCGCGCAATGCCAGCTACAGGCGAG
AACTTCCCAGTCGTGCTGGTGGTACAAGAGATTTTTGGGGTCCATGAGCACATTCAGGACGT

TTGCCGTCGTTTCGCGAAGTTGGGTTTCGTCGCAATCGCTTCAGAACTCTTCGCACGTCAGGG
CGATCCGACGCAACTCGGTAACTCACAAGAAATTATCTCTAAGATTGTCTCAAAAGTTCCTG
ACGCGCAGGTCATGTCAGACCTCGATGCAACTGTAGCCTGGGCTGGTAAATCAAGTAAAGG
GAATCTCAATAAACTTGCGATTACGGGCTTTTGTTGGGGTGGGCGCATTGTATGGCTCTACG
CTGCTCATAACCCACAGGTCAAGGCAGGTGTGGCATGGTATGGCCGTCTTGTCGGGGACGTT
ACGCCGCTCACCCCAAAGCATCCTGTGGATATCGCTTCGGAGCTCAAGACGCCAATCTTAGG
TCTCTACGGGGGCTCCGATGACGGGATTCCAGTTGCTACTGTTGAGCAGATGCGCGACCGTC
TGAAGGCGGGTCGGAGCGGCAGTGAGATTATTGTGTATCCGGACACGCCTCACGCTTTCTTC
GCGGACTACCGCCCTTCCTACCGGAAAGAGCAGGCTGAAGATGGCTGGAAACGTCTCCAGG
CGTGGTTCGATGTTAAGATTTCTTTGGCCGACACCCTGCTCTTCTTCTAGTAATAAGTCGACC
GGCTGCTAACAAAGCCCGAAAGGAAGCTGAGTTGGCTGCTGCCACCGCTGAGCAATAACTA
GCATAACCCCTTGGGGCCTCTAAACGGGTCTTGAGGGGTTTTTTGCTGAAAGCGAGACTAAG
CTTTAAACTTCGGGTCATAGCTGTTTCCTG

**Enzyme 12**

**Alpha/beta hydrolase (unknown substrate) -** *Roseomonas sp.* **KE2513 (Bacteria-)** - Truck

# Amino Acids

>TRINITY_DN50187_c0_g1_i1.p1

MKRRIRITALAVLAAMGLAGPAQAQVAPSHVPLGAAQGLFYRPEGRNAHVAFLIVHRTSDYLR
HIGCTELPRRGFAALCMNTRFVNNELLVDWDRIALDVKEGVAFLRRQPGIRSIVLLGHSGGGPTL
SFYQAVAEAGTAFCQDPRKLVPCRDDLAGLPSADALILADAHPGVPVILLRSLNGAVLDETPGRF
DPGLDPYKPANGYDPNGSSRYTAEFQARYYAAQSARMNRLTDDVLARRQRIAEGRGPYPDNDI
VVIPHGGNPGPGPGGASQLHSLDTAAPLRRTMRPQQLLRNDGSILRQIVQSVSPPELDTFATTNSF
DRGTKLLSIRSYLSTQAVRSINSLDGIDHCSSNNSTVCAVGSISKPLLIMGMTGYLFIRDSEEEFEA
ARSADKDLVFIEGATHGFTPCRTCDANPDAFQNSVRNMFDYIARWVGQRFPG*

# DNA

GTAAAACGACGGCCAGTAGCGCTATTAAAGCTTCGAAATTAATACGACTCACTATAGGGAG
ACCACAACGGTTTCCCTCTAGAAATAATTTTGTTTAACTTTAAGAAGGAGATATACATATGA
TGAAACGGCGGATTCGCATCACAGCATTGGCGGTACTCGCCGCTATGGGTCTTGCCGGCCCG
GCACAGGCGCAGGTCGCTCCAAGCCATGTGCCTCTCGGCGCCGCTCAAGGGTTGTTTTACCG
CCCAGAAGGCCGTAATGCGCACGTGGCGTTCTTGATTGTTCACCGTACTTCTGACTATCTGCG
GCATATTGGCTGCACTGAGCTGCCGCGTCGTGGCTTTGCTGCGTTATGCATGAATACTCGGTT
CGTGAACAACGAACTCCTCGTTGATTGGGACCGGATCGCCCTGGATGTAAAAGAGGGTGTA
GCTTTCCTTCGCCGCCAGCCTGGGATCCGTTCGATCGTTCTTTTGGGTCATTCAGGGGGCGG
CCTACCCTGTCGTTCTACCAAGCGGTGGCTGAGGCCGGCACCGCATTCTGCCAGGATCCTCG
TAAACTCGTTCCGTGCCGTGACGACTTAGCGGGTTTGCCGTCAGCCGACGCGTTAATCCTCG
CTGATGCTCACCCGGGCGTACCTGTAATTTTACTCCGCAGTTTAAACGGGGCTGTGCTGGAT
GAAACACCAGGCCGCTTCGATCCGGGGTTGGACCCATATAAGCCTGCTAACGGCTATGACCC
GAATGGCTCTAGCCGCTACACGGCTGAATTTCAGGCACGGTACTACGCCGCACAGTCGGCAC
GTATGAACCGGCTGACCGACGACGTACTGGCTCGGCGTCAACGGATTGCTGAAGGCCGCGG
TCCATACCCGGATAATGATATCGTTGTTATTCCTCACGGGGGGAATCCAGGGCCTGGGCCTG
GGGGCGCGTCGCAGCTGCACTCCTTGGATACTGCGGCTCCGCTGCGTCGTACGATGCGTCCT

CAGCAGCTGCTTCGCAATGATGGGTCAATTCTGCGCCAAATCGTTCAGAGCGTATCACCTCC
GGAACTGGATACATTCGCTACTACTAATAGTTTCGATCGGGGTACAAAATTGTTGTCTATTC
GGAGTTATTTAAGCACGCAGGCAGTACGTTCTATTAACTCCTTGGATGGTATTGATCACTGT
AGCTCCAACAATTCTACTGTGTGTGCCGTAGGTTCGATTTCGAAGCCTCTTCTCATTATGGGG
ATGACAGGGTACCTCTTTATTCGTGATAGCGAGGAAGAGTTCGAGGCAGCCCGTTCCGCGGA
TAAGGACCTTGTATTTATTGAGGGTGCTACTCATGGGTTCACGCCTTGTCGTACATGTGACGC
GAATCCTGACGCGTTCCAAAACAGCGTCCGCAACATGTTCGATTATATCGCTCGCTGGGTCG
GGCAACGTTTCCCGGGGTAATAATAAGTCGACCGGCTGCTAACAAAGCCCGAAAGGAAGCT
GAGTTGGCTGCTGCCACCGCTGAGCAATAACTAGCATAACCCCTTGGGGCCTCTAAACGGGT
CTTGAGGGGTTTTTTGCTGAAAGCGAGACTAAGCTTTAAACTTCGGGTCATAGCTGTTTCCTG

**Enzyme 13**

**Cutinase (#1) -** *Talaromyces islandicus* **(Fungi)** - Aircraft

# Amino Acids

>TRINITY_DN20387_c0_g3_i3.p1 type:complete len:206 gc:universal
TRINITY_DN20387_c0_g3_i3:1513-896(-)

MPSFTTGALLLLSVLDIASAAAIASAPFQPPGQGNVPVATPTGALCAGQTPTTTAAPTNLPSAVA
GCSNIQLTGTNDTRNDILDGICKPNTLIFARGTFEDPNLGNIVGPPFVAALEDVFGAGNLAVQGV
NDYPAGDVDYCLGGSLTGAENLASVRQYTRSTTQETQLTNLSSSSSKPCPSVPTPSSPSAATAKA
AKSCTTVSRF*

# DNA

GTAAAACGACGGCCAGTAGCGCTATTAAAGCTTCGAAATTAATACGACTCACTATAGGGAG
ACCACAACGGTTTCCCTCTAGAAATAATTTTGTTTAACTTTAAGAAGGAGATATACATATGA
TGCCTTCATTTACTACAGGCGCGTTACTGTTACTCAGTGTACTGGATATTGCTTCCGCGGCTG
CCATCGCTAGTGCCCCATTTCAGCCTCCAGGGCAAGGCAACGTGCCAGTCGCAACTCCGACA
GGCGCTCTTTGTGCTGGTCAAACGCCGACGACAACCGCTGCTCCTACCAATTTGCCGTCAGC
AGTCGCAGGGTGTTCGAATATTCAACTCACGGGGACCAACGATACCCGGAATGACATTTTGG
ACGGGATTTGTAAGCCGAACACGTTAATCTTTGCCCGCGGCACTTTTGAAGATCCAAACCTT
GGGAATATTGTAGGCCCTCCATTCGTGGCAGCTCTGGAGGACGTATTTGGCGCTGGGAACCT
CGCGGTGCAGGGTGTAAACGACTACCCTGCCGGGGATGTTGATTATTGCCTCGGGGGTAGCC
TTACGGGGGCCGAAAACTTAGCTTCAGTGCGCCAATATACCCGTTCGACCACGCAGGAAAC
ACAATTGACCAACCTCTCGAGTTCTTCGTCGAAACCATGTCCTTCCGTGCCAACACCAAGCT
CACCGTCCGCGGCCACAGCCAAGGCTGCGAAATCTTGCACCACCGTGTCCCGTTTCTGATAA
TAAGTCGACCGGCTGCTAACAAAGCCCGAAAGGAAGCTGAGTTGGCTGCTGCCACCGCTGA
GCAATAACTAGCATAACCCCTTGGGGCCTCTAAACGGGTCTTGAGGGGTTTTTTGCTGAAAG
CGAGACTAAGCTTTAAACTTCGGGTCATAGCTGTTTCCTG

# Supplement 2. Calibration Curve for sfGFP

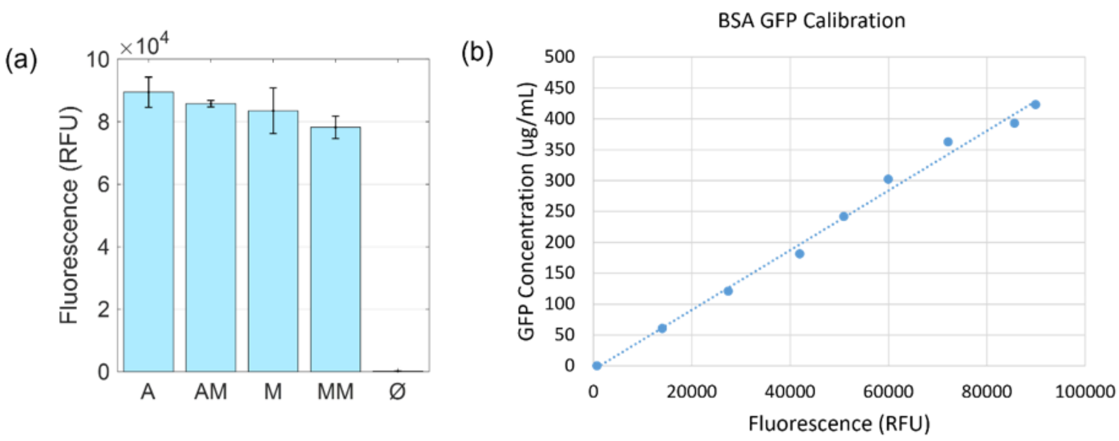


*Figure S1 – (a) End point fluorescence (RFU) of the CFE reactions to produce sfGFP reporter protein with standard deviation (n = 5); A, AM, M, and MM stand for automated transfer, automated transfer and mix, manual transfer, and manual transfer and mix, respectively. (b) Calibration curve of sfGFP RFU to mass concentration determined by adding purified sfGFP (quantified by Pierce 660 nm assay (Thermo Fisher)) in CFPS reaction mixture and measuring the RFU on plate reader at same gain setting as experiments. The linear regression gave an equation of y = 0.0048x - 6.1036 where x is the RFU value and y is the sfGFP concentration. The R2 value for the regression was 0.993.*

# Supplement 3. Test of 13 enzymes with 4-Nitrophenyl Hexanoate substrate

Figure shows the result of large panel test of 13 enzymes using Opentrons. We used 4-nitrophenyl hexanoate as substrate for these enzymes. In the figure, the difference between absorbance after instant scan and initial scan of 13 enzymes at 405 nm absorbance is plotted. From the figure, we can say, enzymes 1-10 and cutinase showed significant cleavage (at 405nm peak) comparing to lysate that had no template.

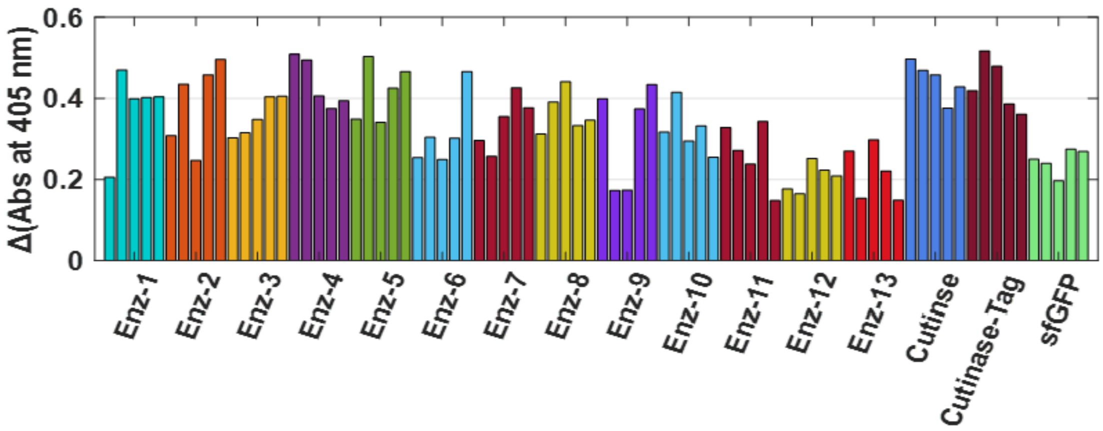


*Figure S2: Barchart showing the result of 13 enzyme test with 4-Nitrophenyl Hexanoate substrate. Each bar shows the difference between absorbance after instant scan and initial scan at 405 nm.*

## Supplement 4. Test of selected 10 enzymes with 4-Nitrophenyl Valerate and 4-Nitrophenyl Hexanoate substrate.

Spectral scan showing the result of the test of selected 10 enzymes with 4-Nitrophenyl Valerate and 4-Nitrophenyl Hexanoate substrate.

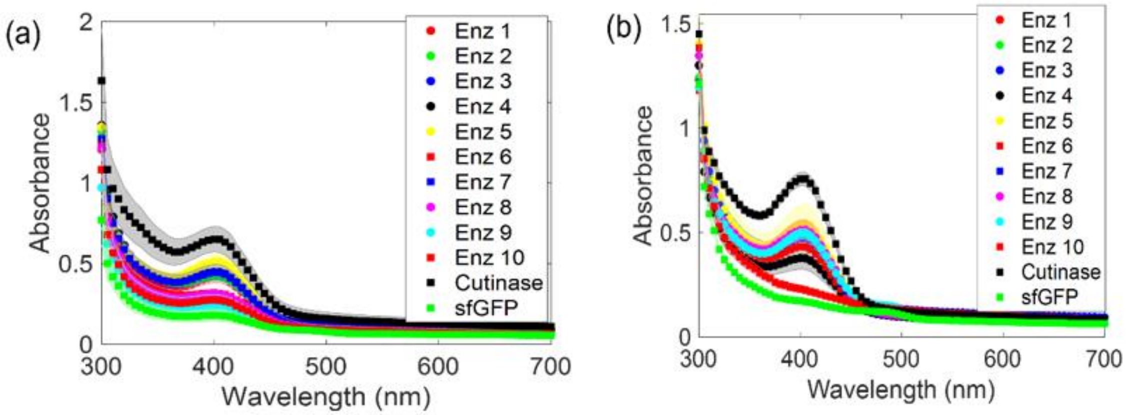


*Figure S3: (a) Spectral scan of dilute lysate with different enzymes after incubating with 4-NPV probe for 15 minutes, showing peak of cleaved 4-NPH probe at 405 nm. (b) Spectral scan of dilute lysate with different enzymes after incubating with 4-NPH probe, instantly showing peak of cleaved 4-NPH probe at 405 nm.*

## Supplement 5. F-test results of automated vs manual experiments

- F test results on accuracy check experiment using mass of 1 μL water that was added manually and with OT-2 using same vs. change tip protocols (Fig 3(a)).

| | h Values | p Values |
|---|---|---|
| A-CT | 0 | 1 |
| A-ST | 1 | 0.0014 |
| M-CT | 1 | 4.33E-05 |
| M-ST | 1 | 4.3E-06 |

- F test results on precision check experiment by measuring absorbance when 1 μL dye was added to 14 μL water (Fig 3(b)).

| | h Values | p Values |
|---|---|---|
| A-CT | 0 | 1 |
| A-ST | 0 | 0.04114 |
| M-CT | 1 | 1.05E-11 |
| M-ST | 1 | 5.30E-12 |

- F test results on end point yield of the CFE reactions done by automated and manual methods using transfer only and transfer and mix protocols (Fig 3(e)).

| | h Values | p Values |
|---|---|---|
| A | 1 | 2.73E-06 |
| AM | 0 | 1 |
| M | 1 | 1.43E-08 |
| MM | 1 | 0.0073 |

- F test results on GFP production rate at first 2.5 hours of the CFE reactions done by automated and manual methods using transfer only and transfer and mix protocols (Fig 3(f)).

| | h Values | p Values |
|---|---|---|
| A | 0 | 0.0083 |
| AM | 0 | 1 |
| M | 0 | 9.15E-07 |
| MM | 1 | 6.57E-09 |

## Supplement 6. T-test on 4-Nitrophenyl Valerate and 4-Nitrophenyl Hexanoate substrate

To measure statistical confidence level of the Nitrophenyl Probe experiment, t-test was done on 11 enzymes against sfGFP both for 4-Nitrophenyl Valerate and 4-Nitrophenyl Hexanoate. A t-test is a statistical test used to compare the means of two groups and determine if there is a significant difference between them. It is particularly useful when the sample sizes are relatively small. The statistical confidence associated with a t-test is typically derived from the p-value. The p-value

represents the probability of observing a t-value as extreme as the one obtained, assuming the null hypothesis (no significant difference) is true.

**Table 2:** t values and p values obtained after t-testing of all enzymes against sfGFP in 4-Nitrophenyl Valerate and 4-Nitrophenyl Hexanoate test

| | **4-Nitrophenyl Valerate** | | **4-Nitrophenyl Hexanoate** | |
|---|---|---|---|---|
| | **t values** | **p values** | **t values** | **p values** |
| **Enzyme - 1** | 7.1999 | 0.0020 | 7.1249 | 0.0020 |
| **Enzyme - 2** | 4.1003 | 0.0148 | 5.5587 | 0.0051 |
| **Enzyme - 3** | 12.6522 | 0.0002 | 9.9637 | 0.0006 |
| **Enzyme - 4** | 16.106 | 0.0001 | 4.7151 | 0.0092 |
| **Enzyme - 5** | 9.9928 | 0.0006 | 6.9209 | 0.0023 |
| **Enzyme - 6** | 9.8712 | 0.0006 | 30.8093 | 0.0000 |
| **Enzyme - 7** | 5.8378 | 0.0043 | 11.6464 | 0.0003 |
| **Enzyme - 8** | 3.5716 | 0.0233 | 6.4122 | 0.003 |
| **Enzyme - 9** | 3.6223 | 0.0223 | 5.4602 | 0.0055 |
| **Enzyme - 10** | 6.1225 | 0.0036 | 3.8463 | 0.0184 |
| **Cutinase** | 7.1618 | 0.0020 | 10.4369 | 0.0005 |

For 4-Nitrophenyl Valerate, observing the t-values and p-values from Table 2, the statistical confidence associated with the observed differences between each enzyme and sfGFP can be assessed. Here, in Table 2 the t-values range from 3.5716 to 16.1060, indicating variability in the magnitude of the observed differences. The larger the absolute value of the t-value, the stronger the evidence against the null hypothesis and the more significant the observed difference. Here, the null hypothesis is there is no significant difference between the means of the sfGFP and corresponding enzyme. The corresponding p-values associated with the t-values are all below 0.05, with values ranging from 0.0001 to 0.0233. A p-value below the chosen significance level (often 0.05) indicates statistical significance and suggests that the observed differences are unlikely to have occurred by chance alone. In Table 2, all the provided p-values for 4-Nitrophenyl Valerate are less than 0.05, indicating statistical significance for each comparison. This implies that there is a high level of confidence (typically associated with a 95% confidence level) that the observed differences between the groups are real and not due to random chance. Therefore, based on the provided t-values and p-values, we can conclude that there is a statistically significant difference between the groups being compared, and there is a high level of confidence in the observed differences.

To understand statistical significance for 4-Nitrophenyl Hexanoate, a t-test was performed for all the enzymes taking sfGFP as base data. The given list of t-values in Table 2 represents the outcomes of 11 separate statistical tests of 11 enzymes, where each t-value corresponds to an independent comparison between that enzyme and sfGFP. It is observed that, the t-values vary widely in magnitude, ranging from 3.8463 to 30.8093, indicating significant variability in the observed differences or effects across the tests. The t-values, like 30.8093 for enzyme-6 and 11.6464 for enzyme 7 and 10.4369 for Cutinase, are notably large, suggesting strong evidence

against the null hypothesis and significant differences or effects between sfGFP and corresponding enzymes. Conversely, smaller t-values, such as 3.8463, for anzyme 10, indicate a lesser degree of difference or effect and may provide less conclusive evidence against the null hypothesis. We also calculated the p-values (Table 2) and found, 10 out of the 11 t-values are associated with p-values less than 0.05, indicating statistical significance at the 95% confidence level. The t-value corresponding to the p-value of 0.0184 (for enzyme 10) is not statistically significant because it is higher than conventional level of confidence of 5%. This statistical significance was shown in figure 5 with asterix marks. The asterix marks are only intended to flag levels of significance for 3 of the most commonly used levels. If a p-value is less than 0.05, it is flagged with one asterix (⋆). If a p-value is less than 0.01, it is flagged with 2 asterix marks (⋆⋆). If a p-value is less than 0.001, it is flagged with three asterix marks (⋆⋆⋆).

## Supplement 7. Observing effect of time with 4-Nitrophenyl Hexanoate probe

The enzymes tested with 4 Nitrophenyl Hexanoate showed instant peak. To see the change with longer exposure time, the absorbance was measured after 1 minute, 15 minutes and 30 minutes. The change in absorbance is shown in the figure S4.

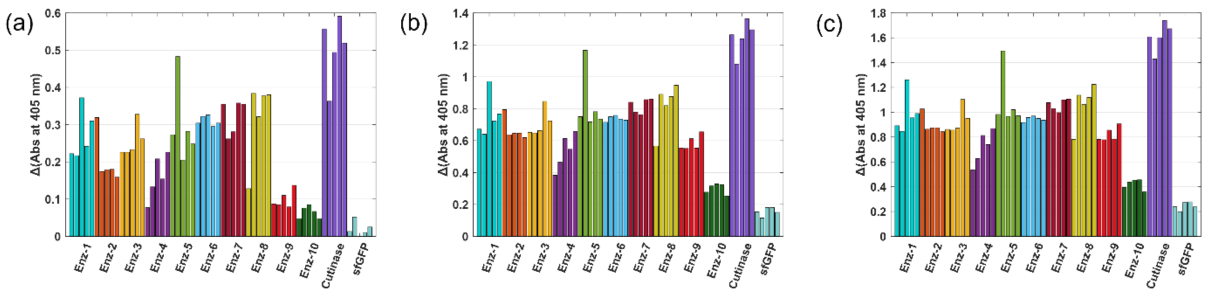


*Figure S4: (a)* Testing putative enzyme panel against 4-Nitrophenyl Hexanoate. Change in absorbance at 405 nm measured after (a) 1 minute (b) 15 minutes (c) 30 minutes incubation with enzyme or control (cutinase as positive and sfGFP as negative).
